# Co-mutation patterns and survival associations in lung cancer: a comparative analysis of Swedish and global cohorts

**DOI:** 10.2340/1651-226X.2026.46330

**Published:** 2026-10-09

**Authors:** Nicholas Karlowatz, Linda Köhn, Cedric Patthey, Jonas Nilsson, Mikael Johansson

**Affiliations:** a Department of Diagnostics and Intervention, Oncology, Umeå University, Umeå, Sweden; b Department of Medical Biosciences, Pathology, Umeå University, Umeå, Sweden

**Keywords:** Non-small-cell lung cancer, co-mutations, cancer genomics, survival analysis

## Abstract

**Background and purpose:**

Co-occurring mutations in lung cancer can influence disease progression and treatment response. Understanding these patterns may refine prognostic stratification and therapeutic decisions.

**Patients/materials and methods:**

We analysed targeted sequencing data from 528 North Swedish non-small cell lung cancer (NSCLC) patients, validating findings in 2,239 global patients. Co-mutation enrichment was assessed using the Fisher’s exact test with odds ratios, and survival associations were evaluated using Kaplan–Meier/log-rank analyses and Cox models.

**Results:**

The North Sweden cohort showed non-random co-mutation patterns shaped by pathway dependencies. The combination of Kirsten rat sarcoma virus oncogene homolog (*KRAS*) and STK11 co-mutation was significantly enriched in both North Sweden (*p* = 4.7 × 10^-4^) and globally (*p* = 2.5 × 10^-22^), with North Sweden co-mutants showing significantly worse survival compared with *KRAS*-only cases (*p* = 0.037). Epidermal growth factor receptor (*EGFR*)+phosphate and tensin homolog (*PTEN*) co-mutation was significantly associated with poorer survival in both cohorts (North Sweden: *p* = 0.013; World: *p* = 0.0013). *EGFR*+TP53 co-mutation trended towards poorer survival compared with *EGFR* alone, reaching significance globally (*p* = 1.4 × 10^-5^). Adenomatous polyposis coli (*APC*) and *PTEN* were positively enriched in North Sweden (*p* = 0.018), and co-mutants had worse survival than *APC*-only cases (*p* = 0.029), a pattern mirrored globally. *KRAS*+TP53 was a frequent co-mutation but not prognostically significant. *KRAS* and *EGFR* were strongly mutually exclusive in both cohorts.

**Interpretation:**

Pathway-level co-mutation interactions, rather than individual mutation burden, drive survival heterogeneity in lung cancer. Specific co-mutations, such as *KRAS*+STK11 and *APC*+*PTEN*, are enriched and linked to worse survival, supporting the clinical relevance of co-mutation analysis in NSCLC.

## Introduction

Advances in genomic profiling show that non-small cell lung cancer (NSCLC) is shaped by networks of interacting mutations, with co-mutations emerging as important determinants of tumour behaviour and patient outcomes [1]. The introduction of next-generation sequencing (NGS) in clinical routine to detect actionable driver mutations or re-arrangements such as Epidermal growth factor receptor (*EGFR*), Kirsten rat sarcoma virus oncogene homolog (*KRAS*), Anaplastic lymphoma kinase (*ALK*), B-Raf proto-oncogene (*BRAF*), Tropomyosin receptor kinase A (*NTRK1-3*), Met proto-oncogene (*MET*), Re-arranged during transfection (*RET*), Erythroblastic oncogene B-2 (*ERBB2*) and ROS proto-oncogene-1 (*ROS1*) has also revealed extensive genomic heterogeneity in lung cancer [2]. Rather than operating separately, multiple mutations often affect shared cellular processes, intensifying oncogenic pathways, diminishing regulatory safeguards and contributing to tumour heterogeneity. Whilst targeted therapies have improved outcomes for patients harbouring specific driver mutations, clinical responses remain variable, suggesting that additional genomic factors may modulate treatment efficacy and prognosis [2, 3].

Recent large-scale cancer genomics studies have highlighted that, despite the vast number of possible combinations, most driver mutations do not co-occur at random. Instead, evolutionary constraints such as functional redundancy, synthetic lethality and negative selection shape the landscape of co-mutations by limiting which combinations persist in tumours, whilst a few ‘positive loops’ of co-mutations are favoured because they provide a selective advantage [4, 5]. For example, some co-mutations are mutually exclusive due to overlapping functions or deleterious effects, whilst others, such as *KRAS* and STK11 in lung cancer, are positively selected and associated with aggressive disease [4]. This evolutionary perspective suggests that the patterns of co-mutation observed in tumours are not only a record of past selective pressures but also a source of clinically relevant dependencies [3, 5].

Beyond the importance of individual druggable driver genes, the pattern of co-occurring mutations carries prognostic information that single-gene analysis can miss [3, 6]. *KRAS*+TP53 co-mutations are linked to aggressive phenotypes, whilst *EGFR* mutations co-occurring with TP53 alterations are associated with inferior responses to tyrosine kinase inhibitors [7, 8]. The PI3K–AKT–mTOR axis is a critical signalling hub frequently altered by mutations in phosphatase and tensin homolog (*PTEN*), PIK3CA and PIK3R1. However, the prognostic significance of co-mutations within this pathway in lung cancer remains incompletely characterised [9, 10]. *MET*-driven signalling can bypass *EGFR* inhibition in tumours harbouring concurrent *EGFR* and *MET* alterations, whilst *PTEN* loss can blunt PI3K-directed strategies by activating alternative survival routes [7, 9]. Certain co-mutation patterns not only affect signalling within tumour cells but also modify the surrounding microenvironment. By altering antigen presentation, cytokine activity, oxidative stress responses and myeloid cell recruitment, these co-mutations can impair immune detection and limit the success of immunotherapy [11, 12]. Together, these findings suggest that co-mutation patterns reflect biologically relevant pathway interactions that influence tumour behaviour and treatment response and merit further study across larger and more diverse cohorts [3, 6].

In this study, we performed an analysis of co-mutation patterns in 528 NSCLC patients from North Sweden, with validation in 2,239 patients from international cohorts. We show that specific co-mutation signatures demonstrate reproducible associations with survival outcomes across independent populations. We characterise the co-mutational landscape in a North Swedish cohort, identify co-mutation pairs with significant survival associations and compare these findings to global NSCLC cohorts.

## Patients/material and methods

### Study population and data sources

The North Sweden cohort included 528 patients with histologically confirmed lung cancer diagnosed in North Sweden between 2010 and 2021 and sequenced on the regional 67-gene Archer VariantPlex^®^ Solid Tumour panel. The vast majority of patients were sequenced at diagnosis prior to any treatment given. Age, sex, smoking history, stage and survival were extracted from regional healthcare databases. Ethical approval was granted by the Swedish Ethical Review Authority (DNR 2021-06549-01).

For validation, we obtained genomic and clinical data from 2,239 lung cancer patients from five independent cBioPortal studies (accessed June 2024): TCGA Lung Adenocarcinoma 2016 (*n* = 925) [13], MSKCC 2020/2023 (*n* = 896) [14], OncoSG Lung Adenocarcinoma (*n* = 234) [15], NCI 2022 Never-Smokers (*n* = 128) [16] and OncoSG 2020 (*n* = 56) [17]. Overall survival was available for 2,197 patients and stage for 1,206. Analyses were restricted to genes in the North Sweden 67-gene panel.

### Mutation data processing

Mutations were annotated using Human Genome Variation Society (HGVS) nomenclature. Analyses included non-synonymous variants (missense, nonsense, frameshift, splice-site and in-frame indels) and excluded synonymous variants and variants of unknown significance.

Mutation-event count was defined as the number of retained non-synonymous events per patient. Patients were grouped as 0, 1, 2, 3, 4, 5 or ≥ 6 events for survival plots. Co-mutation required non-synonymous mutations in both genes of a pair at variant allele frequency ≥ 5%.

### Co-mutation enrichment analysis

Co-mutation enrichment was assessed for all gene pairs in the North Sweden and World cohorts using the Fisher’s exact test. For each gene pair, a 2 × 2 contingency table was constructed to compare observed versus expected co-occurrence frequencies under the assumption of independence. Odds ratios and lift quantified enrichment direction and magnitude; odds ratio (OR) > 1 indicated co-occurrence above chance, and OR < 1 indicated mutual exclusivity. Nominal two-sided *p* < 0.05 was used to flag pairs as significant, with false discovery rate (FDR)-adjusted *q*-values reported in Supplementary Table 5. Pairs required at least five co-mutated patients in North Sweden or 12 in the World cohort and were visualised as signed significance heatmaps.

### Survival-associated co-mutation analysis

For the co-mutation survival screen, patients were grouped as both mutated, gene A only or gene B only. Overall survival was time from diagnosis to death from any cause or last follow-up. For each pair, log-rank tests compared double-mutant cases with each single-gene group, and the smaller *p*-value was used as the screening metric.

For selected pairs, curves compared gene A only, gene B only, double-mutant and all-patient groups, with log-rank testing of double-mutant versus single-mutant groups. Cohort-level overall survival was summarised as Kaplan–Meier median Overall Survival (OS), with follow-up estimated by reverse Kaplan–Meier to avoid bias from short censoring times.

Cox proportional hazards models estimated hazard ratios (HRs) with 95% confidence intervals for mutated versus non-mutated status for each gene or gene pair. Two-sided Wald tests provided *p*-values, and forest plots summarised relative risk across cohorts. For the principal co-mutation pairs, multivariable Cox models additionally adjusted for age, sex, smoking history and stage.

### Statistical analysis

Analyses were performed in Python using pandas, lifelines, scipy.stats, matplotlib and seaborn. Two-sided *p* < 0.05 defined statistical significance for prespecified analyses; the pairwise co-mution enrichment screen was exploratory, with Benjamini–Hochberg FDR-adjusted *q*-values reported alongside.

## Results

### Patient characteristics and cohort composition

The North Sweden cohort comprised 528 NSCLC patients (Table 1), with a median age of 72 years. The mean number of mutation events per patient was 2.74. Mutation-event burden was distributed as follows: 0–1 events in 30% of patients, 2–3 in 44%, 4–5 in 20% and 6 or more in 7% (Figure 1A). At diagnosis, 139 patients (26%) were stage I, 44 (8%) stage II, 101 (19%) stage III and 244 (46%) stage IV. The validation (World) cohort included 2,239 patients, of whom 2,197 had survival data available. Stage at diagnosis was available for 1,206 patients: 600 (50%) were stage I, 313 (26%) stage II, 243 (20%) stage III and 50 (4%) stage IV. The median age amongst patients with recorded age was 67 years. The mean number of mutations per patient was 2.58. Mutation-event burden was distributed as follows: 0–1 events in 26% of patients, 2–3 in 53%, 4–5 in 16% and 6 or more in 5% (Figure 1A; Table 1). Cohort-level survival summaries are provided in Supplementary Table 1 as Kaplan–Meier median OS together with reverse Kaplan–Meier median follow-up. Although the exact bin frequencies differed modestly between cohorts, both distributions were centred on the 2–3 mutation-event groups.

**Table 1:** Cohort characteristics of the North Sweden and World NSCLC cohorts.

Characteristic	North Sweden (*n* = 528)	World (*n* = 2,239; OS available *n* = 2,197)
Total patients	528	2,239
Adenocarcinoma, *n* (%)	399 (75.6%)	1,783 (79.6%)
Squamous cell carcinoma, *n* (%)	100 (18.9%)	456 (20.4%)
Median age, years	72 (available *n* = 528)	67 (available *n* = 1,307)
Current smoker, *n* (%)	196 (37.1%)	231 (19.9%; available *n* = 1,161)
Former smoker, *n* (%)	256 (48.5%)	799 (68.8%; available *n* = 1,161)
Mean mutation events per patient	2.74 (range 0–44)	2.58 (range 0–14)
0–1 mutation events	156 (29.5%)	585 (26.1%)
2–3 mutation events	231 (43.8%)	1,184 (52.9%)
4–5 mutation events	104 (19.7%)	354 (15.8%)
≥ 6 mutation events	37 (7.0%)	116 (5.2%)
Stage I	139 (26.3%)	600 (26.8%)
Stage II	44 (8.3%)	313 (14.0%)
Stage III	101 (19.1%)	243 (10.9%)
Stage IV	244 (46.2%)	50 (2.2%)
Stage missing	0 (0%)	1,033 (46.1%)

**Figure 1:**
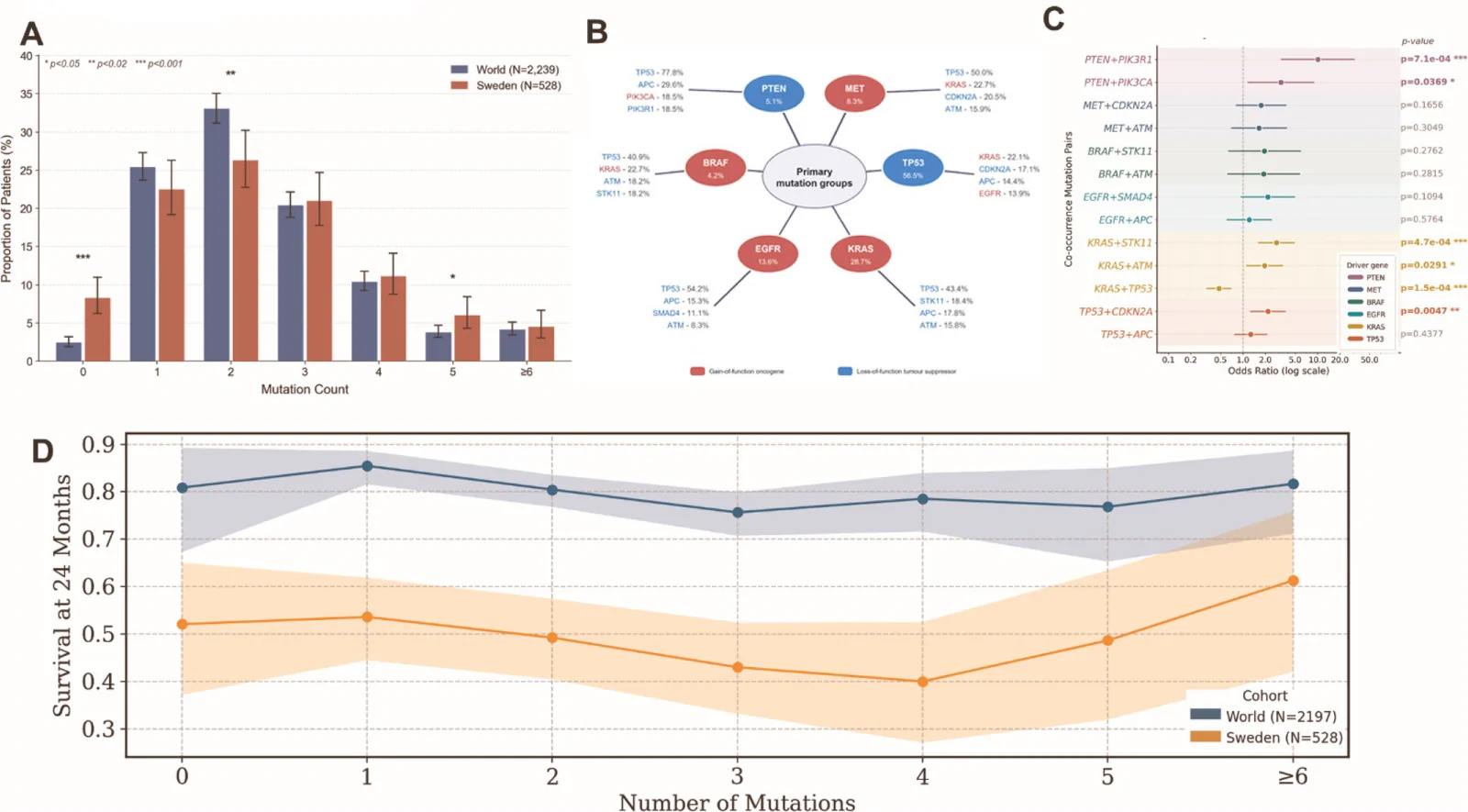
(A) Frequency distribution of mutation-event counts (0, 1, 2, 3, 4, 5 and ≥ 6) in the World (blue, *n* = 2,239) and North Sweden (red, *n* = 528) cohorts. Error bars indicate the standard deviation of the binomial proportion. Asterisks denote χ^2^ test significance for between-cohort differences within each mutation-count group (*** *p* < 0.001, ** *p* < 0.01, * *p* < 0.05, ns not significant). (B) Primary Mutation groups and co-mutation patterns in the cohort. Each central node shows the frequency of the indicated mutation group, with colours distinguishing gain-of-function oncogenes from loss-of-function tumour suppressor genes. Adjacent lists show the four most frequent co-mutated genes within each group, ranked by frequency and colour-coded by functional class. (C) Forest plot of top co-mutations amongst six key genes in the North Sweden cohort (*n* = 528). Each point represents a top co-mutated gene pair. Odds ratios (log scale; OR > 1 indicates co-occurrence above chance, OR < 1 mutual exclusivity) and 95% confidence intervals are shown. *P*-values are annotated with asterisks (*p < 0.05, **p < 0.01, ***p < 0.001). (D) Twenty-four-month overall survival stratified by mutation-count group in the World (blue) and North Sweden (orange) cohorts. Shaded areas indicate 95% confidence intervals.

The most frequently mutated genes in the North Sweden cohort were TP53 (56%), *KRAS* (29%), *EGFR* (14%), STK11 (11%), *MET* (8%) and *PTEN* (5%). The frequency of *EGFR* and TP53 co-mutation was 7%. In the World cohort, the most common mutations were TP53 (54%), *KRAS* (26%), *EGFR* (26%), STK11 (12%), *MET* (4%) and *PTEN* (4%), with *EGFR* and TP53 co-mutation in 12% of patients (Figure 1B).

### Co-mutation enrichment patterns

Co-mutation profiling in North Sweden revealed several dominant, biologically coherent pairings amongst *MET*, TP53, *KRAS*, *EGFR*, *BRAF* and *PTEN* (Figure 1B). A notable pair, *KRAS*+TP53, was significantly underrepresented (Sweden OR = 0.48, *p* = 1.5 × 10^-4^), a pattern echoed globally (World OR = 0.31, *p* = 3.5 × 10^-32^), supporting their mutual exclusivity across populations (Figure 1B, C). *KRAS* preferentially co-occurred with STK11 in both the North Sweden (OR = 2.81, *p* = 4.7 × 10^-4^) and World cohort (OR = 3.78, *p* = 2.5 × 10^-22^), highlighting a robust molecular subtype. *KRAS* also showed positive enrichment with ATM (OR = 1.95, *p* = 0.029) and SMAD4 (OR = 2.18, *p* = 0.045) in North Sweden, though these were not significant globally. *KRAS*+*EGFR* co-mutation was extremely rare in both cohorts and significantly depleted in North Sweden (OR = 0.16, *p* = 2.2 × 10^-6^) and globally (OR = 0.027, *p* = 9.8 × 10^-75^), confirming strong mutual exclusivity.

*EGFR* mutations most often paired with TP53 in North Sweden (OR = 0.90, *p* = 0.70), but this was not positively enriched, and globally, *EGFR*+TP53 co-mutation was significantly depleted (OR = 0.64, *p* = 5.3 × 10^-6^), again supporting mutual exclusivity. *EGFR*+*BRAF* co-mutation was absent in North Sweden (OR = 0.00, *p* = 0.057) and depleted globally (OR = 0.31, *p* = 1.4 × 10^-5^). *BRAF*+*KRAS* co-mutation was not significant in North Sweden (OR = 0.72, *p* = 0.64) but was significantly depleted globally (OR = 0.29, *p* = 4.7 × 10^-6^).

*MET* most frequently co-mutated with TP53 and *KRAS* in North Sweden, but both pairs trended towards negative enrichment (*MET*+TP53 OR = 0.75, *p* = 0.43; *MET*+*KRAS* OR = 0.71, *p* = 0.39). In the World cohort, both *MET*–TP53 (OR = 0.58, *p* = 0.012) and *MET*–*KRAS* (OR = 0.44, *p* = 0.0062) were significantly depleted, supporting a global pattern of mutual exclusivity. *PTEN* showed strong positive associations in Sweden with TP53 (OR = 2.83, *p* = 0.027), PIK3CA (OR = 3.22, *p* = 0.037) and especially PIK3R1 (OR = 10.1, *p* = 0.00071), indicating a tendency for *PTEN* loss to co-occur with PI3K pathway alterations. In the World cohort, *PTEN*+TP53 was also significantly enriched (OR = 2.78, *p* = 1.9 × 10^-5^), but *PTEN*+PIK3CA and *PTEN*+PIK3R1 were not significant. For TP53, the most notable co-mutation in Sweden was with CDKN2A (OR = 2.17, *p* = 0.0047), which was also significant globally (OR = 3.20, *p* = 6.5 × 10^-10^). After the Benjamini–Hochberg correction, the strongest signals remained significant in both cohorts (*q* < 0.05), including *KRAS*+*EGFR* and *KRAS*+TP53 mutual exclusivity, *KRAS*+STK11, *PTEN*+PIK3R1 and TP53+CDKN2A in North Sweden; several weaker North Sweden pairs did retain significance after correction, whereas all reported World cohort enrichments did (Supplementary Table 5).

### Survival associations with mutation burden

Twenty-four-month overall survival varied only modestly across mutation-count groups (Figure 1D). Patients in both cohorts were concentrated in the 1–3 mutation-event groups, whereas the ≥ 6 group remained comparatively small. Across corresponding mutation-count groups, the World cohort consistently showed higher 24-month survival than the North Sweden cohort, likely reflecting the much higher proportion of stage IV disease in the North Sweden series. Within each cohort, however, no clear relationship between mutation-event burden and survival was evident.

### Co-mutation associations with survival

To systematically identify co-mutation pairs with survival relevance, we performed a pairwise screen across the 67 genes in both cohorts, plotting co-mutation frequency against pairwise log-rank survival difference for all screened gene combinations (Figure 2A, North Sweden; Figure 2D, World). The screen highlighted several recurrent pairs with strong survival differences between co-mutated and single-gene groups, notably *EGFR*+TP53, *EGFR*+*PTEN*, *KRAS*+STK11 and TP53+RB1 (Supplementary Figures 3F–G).

**Figure 2:**
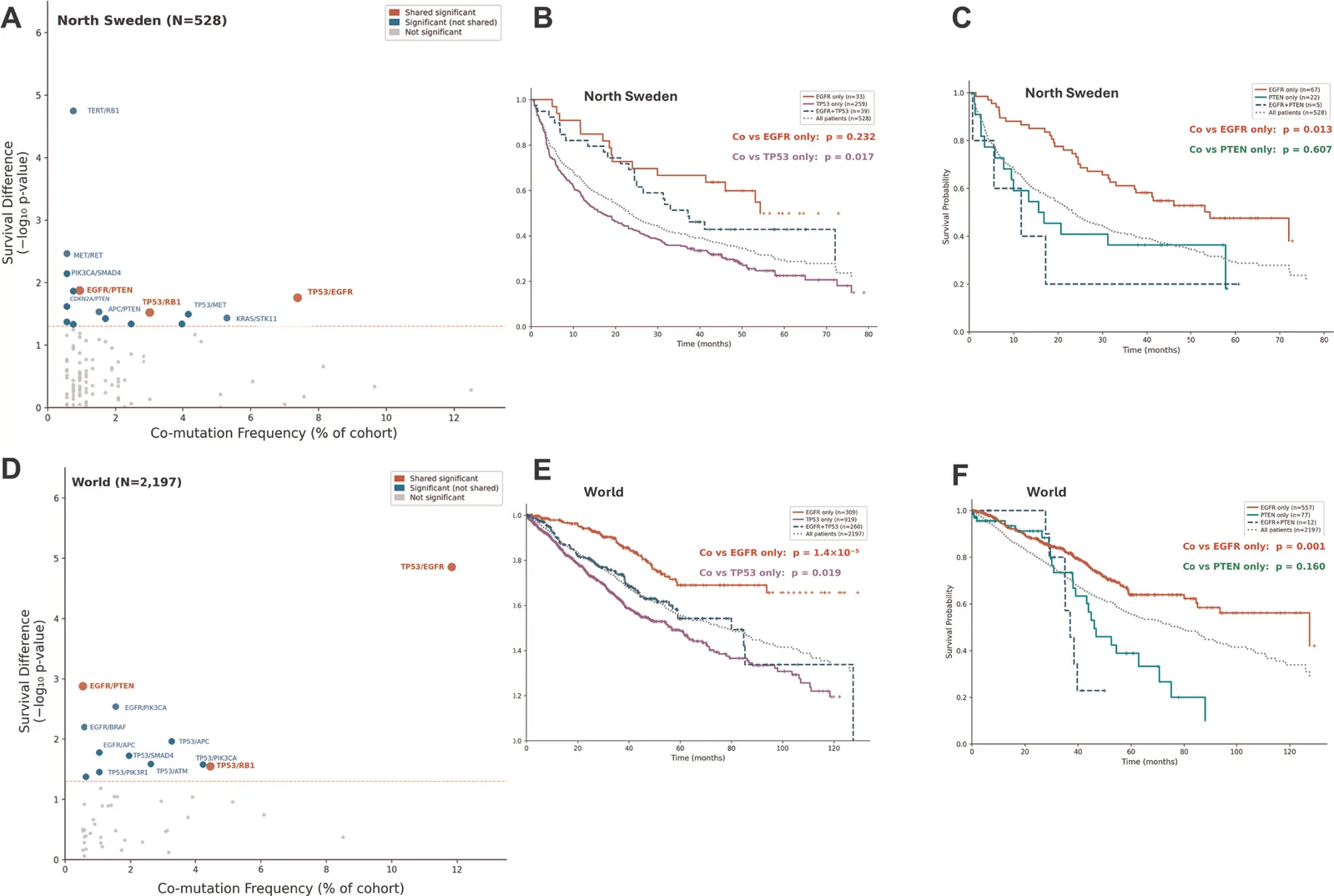
(A, D) Cohort-level co-mutation screening scatter plots showing gene-pair co-mutation frequency versus −log10(p) from log-rank tests in the North Sweden (A) and World (D) cohorts. The dashed line indicates p = 0.05. Points are coloured by significance in both cohorts (red), one cohort (blue) or neither (grey). (B–C, E–F) Kaplan–Meier estimates of overall survival stratified by co-mutation status in EGFR and TP53 (B, E) or EGFR and PTEN (C, F) in the North Sweden (B–C) and World (E–F) cohorts.

Considering *EGFR’s* favourable prognostic association, we next assessed genes that could modify this effect when co-mutated with *EGFR*. Across both cohorts, *EGFR*+*PTEN* co-mutation was associated with poorer overall survival compared with *EGFR*-only cases (North Sweden: *n* = 5, *p* = 0.013; World: *n* = 12, *p* = 0.0013), consistent with *EGFR*–PI3K/AKT pathway cross-talk (Figure 2B, E). *EGFR*+TP53 showed a more complex pattern: in North Sweden, co-mutants did not differ significantly from *EGFR*-only cases (*n* = 39, *p* = 0.23), whereas in the World cohort, co-mutants had poorer survival than *EGFR*-only cases (*n* = 260, *p* = 1.4 × 10^-5^) (Figure 2C, F).

Whilst *KRAS*+TP53 was a common co-mutation (World: *n* = 187 with OS data, North Sweden: *n* = 66), it was not prognostically significant in either cohort on detailed pairwise Kaplan–Meier analysis (Supplementary Figure 1C–D). By contrast, *MET*-linked pairs showed cohort-specific behaviour: *MET*+TP53 showed the strongest survival separation in North Sweden, whilst *APC*-linked patterns were more pronounced in the World cohort.

TP53-associated adverse prognosis was heterogeneous across co-mutation contexts. In the North Sweden cohort, RB1+TP53 co-mutants had worse survival than TP53-only cases (*p* = 0.030), and *MET*+TP53 co-mutants had worse survival than *MET*-only cases (*p* = 0.032) with borderline separation from TP53-only cases (*p* = 0.052) (Supplementary Figure 3G; Figure 3D). In the World cohort, RB1+TP53 was strongly enriched and showed a nominal difference versus RB1-only cases (*p* = 0.028) but not versus TP53-only cases (*p* = 0.77), whereas *MET*+TP53 did not show a robust pairwise survival difference on detailed Kaplan–Meier analysis (Supplementary Figure 3F and Supplementary Figure 2A).

**Figure 3:**
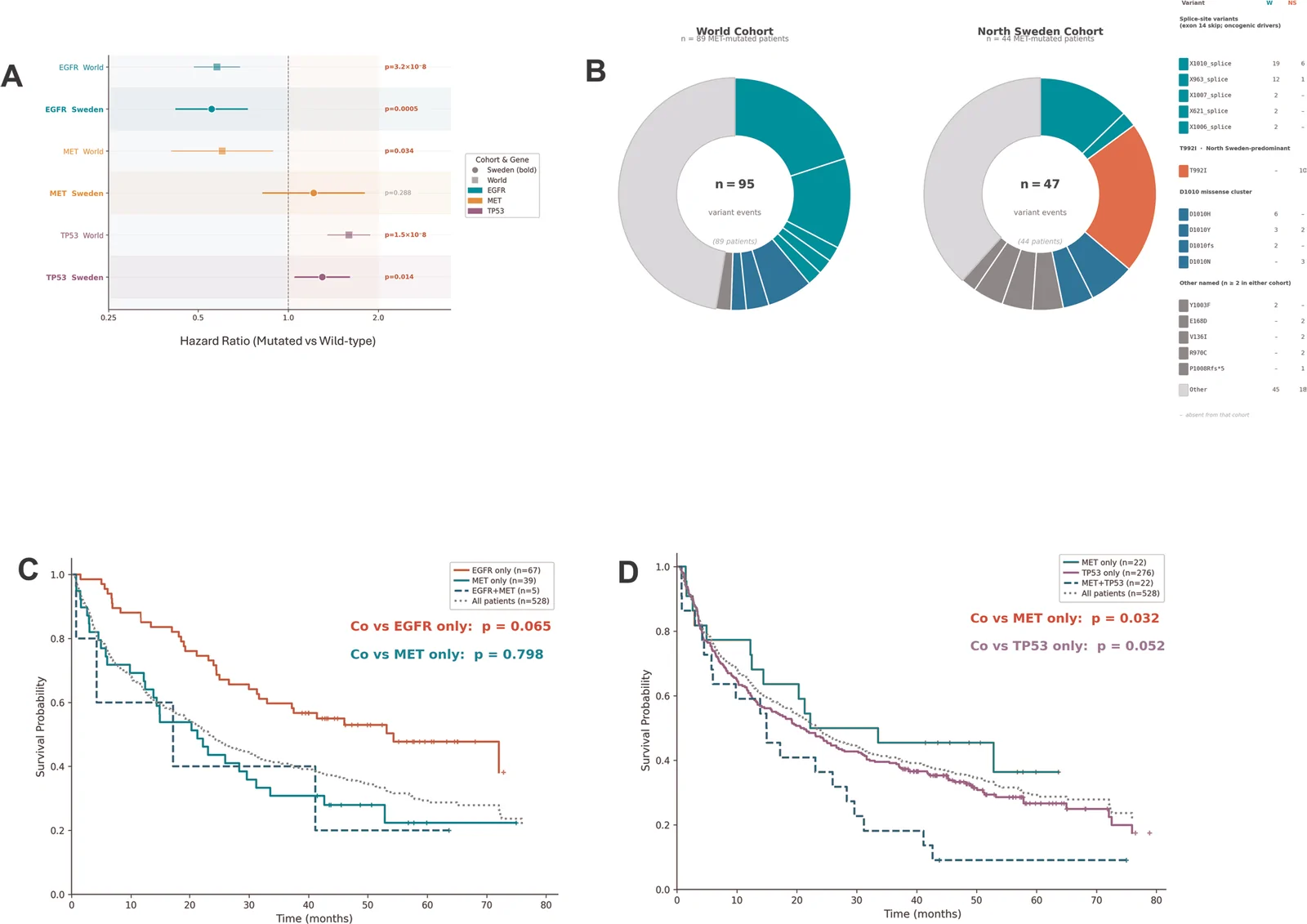
(A) Unadjusted univariable Cox proportional hazards models for overall survival. Forest plot shows hazard ratios (HRs) with 95% confidence intervals for EGFR (teal), MET (orange) and TP53 (purple) mutation status. (B) Distribution of MET protein-altering variants across the World and North Sweden cohorts. (C–D) Kaplan–Meier estimates of overall survival in the North Sweden cohort stratified by co-mutation status in MET and EGFR (C) or MET and TP53 (D).

Amongst the major driver genes, *MET* showed divergent prognostic patterns between cohorts. *EGFR* mutations were associated with a significantly reduced hazard in both the World cohort (HR = 0.58, *p* < 1 × 10^-6^) and North Sweden (HR = 0.55, *p* = 0.0006), indicating a protective effect (Figure 3A). In contrast, TP53 mutations were associated with a significantly increased hazard (World: HR = 1.60, *p* < 1 × 10^-6^; North Sweden: HR = 1.30, *p* = 0.015), consistent with their established negative prognostic role. *MET* mutations were associated with reduced hazard in the World cohort (HR = 0.59, *p* = 0.036) but showed a non-significant trend towards increased hazard in North Sweden (HR = 1.21, *p* = 0.29) (Figure 3A; Supplementary Table 3).

The divergent *MET* signal was accompanied by a different mutation spectrum in the two cohorts: the North Sweden cohort was dominated by p.Thr992Ile (*n* = 10) and X1010_splice (*n* = 6), whereas the World cohort was characterised by X1010_splice (*n* = 19), X963_splice (*n* = 12) and p.Asp1010His (*n* = 6) variants (Figure 3B; Supplementary Table 4). Kaplan–Meier analyses further showed that the adverse *MET* pattern in Sweden was most evident in specific co-mutation contexts. *MET*+TP53 co-mutants had worse survival than *MET*-only cases (*p* = 0.032) and showed borderline separation from TP53-only cases (*p* = 0.052), whereas *MET*+*EGFR* showed only a trend towards poorer survival compared with MET-only cases (*p* = 0.065) (Figure 3C).

### Enriched co-mutations and survival

To further study enriched co-mutations, we linked enrichment analysis with pairwise survival analysis to examine whether recurrent selection also tracked with adverse outcome (Figure 4A, B). *KRAS*+STK11 was the strongest reproducibly enriched pair (North Sweden: OR = 2.81, *p* = 4.7 × 10^-4^; World: OR = 3.78, *p* = 2.5 × 10^-22^; 28 and 136 co-mutants, respectively), and North Sweden co-mutants showed worse survival than *KRAS*-only cases (*p* = 0.037), whereas the World cohort showed the same direction without statistical significance (*p* = 0.18) (Figure 4C; Supplementary Figure 3A). Adenomatous polyposis coli (*APC*)+*PTEN* was positively enriched in North Sweden (OR = 2.98, *p* = 0.018; 8 co-mutants) but not in the World cohort; nevertheless, co-mutants had worse survival than *APC*-only cases in both North Sweden (*p* = 0.029) and the World cohort (*p* = 0.033), and in the World cohort also differed from *PTEN*-only cases (*p* = 0.0020) (Figure 4D; Supplementary Figure 3B). PIK3R1+*PTEN* showed strong enrichment in North Sweden (OR = 10.1, *p* = 7.1 × 10^-4^; 5 co-mutants) but not in the World cohort, and the associated survival differences remained non-significant in both cohorts, consistent with limited power (Figure 4E; Supplementary Figure 3C).

**Figure 4:**
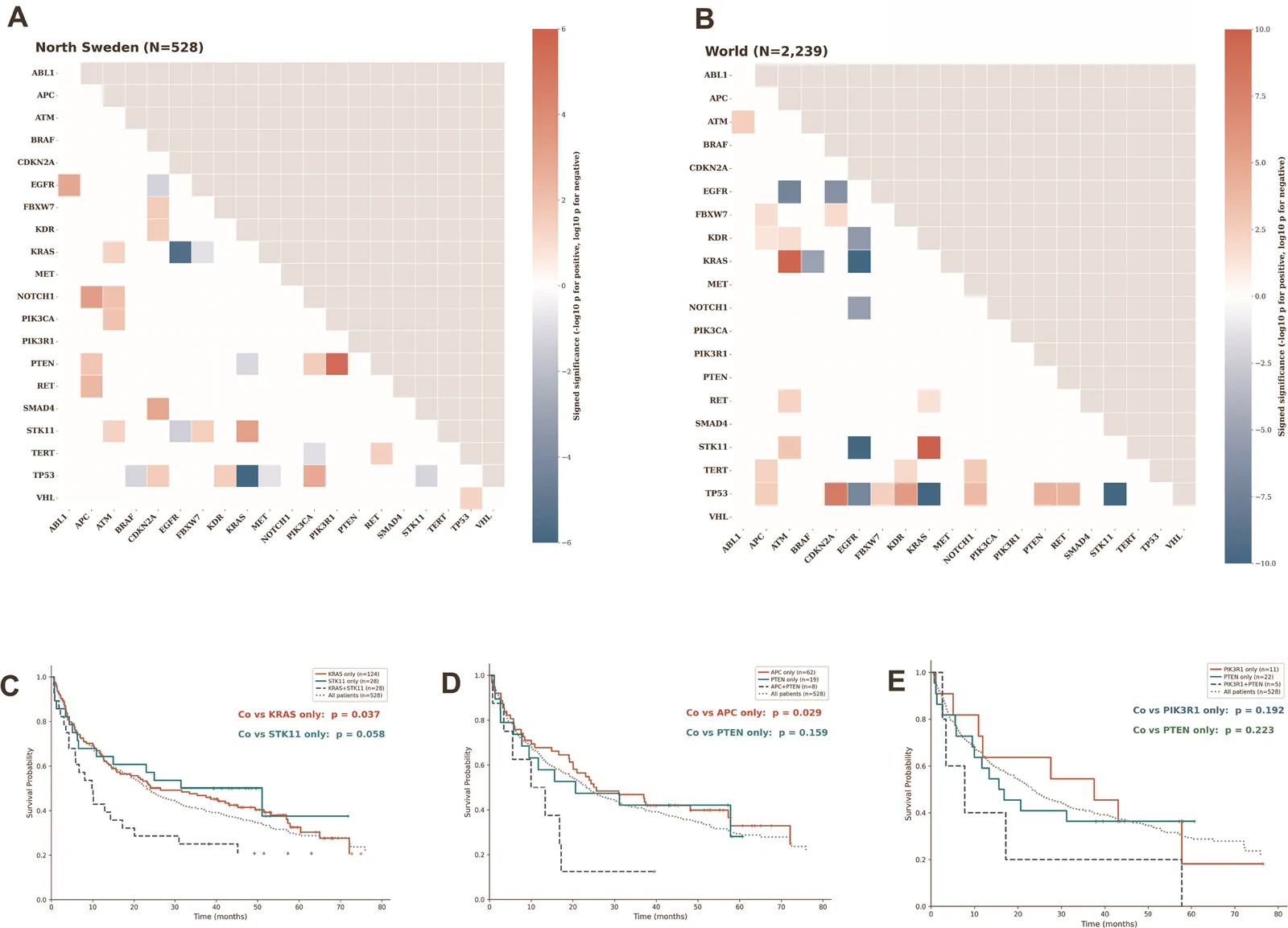
(A–B) Co-mutation enrichment heatmaps for pairs of panel genes in the North Sweden (A) and World (B) cohorts. Each cell represents one gene pair tested with Fisher’s exact test. Cell colour shows signed significance: –log10(p) for positive enrichment (red) and log10(p) for mutual exclusivity (blue), with darker shades indicating stronger significance and white indicating no enrichment. Grey cells mark the redundant upper triangle. (C–E) Kaplan–Meier estimates of overall survival stratified by co-mutation status for KRAS and STK11 (C), APC and PTEN (D) and PIK3R1 and PTEN (E) in the North Sweden cohort.

CDKN2A+FBXW7 was positively enriched in both cohorts (North Sweden: *p* = 0.031, 6 co-mutants; World: *p* = 0.022, 8 co-mutants) and showed a non-significant trend towards worse survival in both cohorts (Supplementary Figures 3J–K).

Amongst negatively enriched pairs, *EGFR*+*KRAS* showed strong mutual exclusivity in both North Sweden (*p* = 2.2 × 10^-6^; 5 co-mutants) and the World cohort (*p* = 9.8 × 10^-75^; 8 co-mutants), with no significant survival difference (Supplementary Figures 3H–I). *EGFR*+CDKN2A was strongly depleted in both cohorts (Sweden *p* = 0.094, World *p* = 1.5 × 10^-7^), with no robust pairwise survival difference (Supplementary Figures 3L–M). *EGFR*+STK11 was similarly depleted (World *p* = 3.8 × 10^-30^), but the co-mutant group was too small for detailed survival analysis.

## Discussion and conclusion

Our comparative analysis of North Sweden and global NSCLC cohorts shows that co-mutation patterns are shaped partly by functional relationships between oncogenic drivers. Although individual pairs may reflect chance overlap in a shared mutational context, a subset departed consistently from expectation across both populations [18]. These non-random pairs clustered at pathway interfaces, where cooperation or redundancy may select for or against co-occurrence.

Mutation burden alone was a poor predictor of 24-month survival, reinforcing that prognosis depends more on which genes are mutated together than on mutation count. The higher survival in the World cohort at each mutation-count level likely reflects fewer late-stage cases rather than intrinsic biological differences. Stage and mutation burden did not track closely, suggesting that they capture distinct aspects of disease biology [19]. Thus, mutation count may affect survival mainly in specific co-mutation contexts [20]. When specific gene pairs are recurrently co-selected, they likely do so because the combination confers a selective advantage by intensifying a shared pathway, disabling a complementary tumour suppressor, or both, rather than arising by chance [21].

The mutual exclusivity of *KRAS* with TP53 and *EGFR* was consistent across cohorts. For *KRAS* and *EGFR*, this likely reflects functional redundancy because both activate MAPK and PI3K/AKT signalling [21, 22]. *KRAS* and TP53 may be mutually exclusive because each can disrupt cell-cycle control and apoptosis, making co-occurrence unnecessary or deleterious [23]. Global depletion of *BRAF*+*KRAS* supported a similar pattern amongst alternative RAS-MAPK drivers [24].

Positive enrichment may reflect cooperation. *KRAS*+STK11, *APC*+*PTEN* and PIK3R1+*PTEN* each illustrate complementary pathway disruption [4, 9]. *KRAS*+STK11 was the most reproducible pair, enriched in both cohorts and associated with poorer survival in North Sweden, with the same direction in the World cohort [4]. Biologically, *KRAS*-driven proliferation combined with STK11 loss can suppress AMPK-dependent energy sensing and STING-mediated interferon signalling, producing an immune-cold phenotype linked to checkpoint inhibitor resistance [4, 25, 26].

*APC*+*PTEN* co-mutation may remove parallel brakes on growth and apoptosis through combined WNT and PI3K/AKT disruption, consistent with enrichment in North Sweden and worse survival in both cohorts [9]. *PTEN* also clustered with PIK3R1 and PIK3CA, supporting accumulation of PI3K-axis alterations [9, 25]. Although numbers were small, these alterations affected about 13% of North Sweden patients and may define a subgroup for future evaluation of PI3K-pathway targeting [9, 10, 27].

Overall, co-mutations appeared most informative when paired alterations complemented rather than duplicated each other. Strengthening pathway output or combining pathway activation with loss of normal control, as seen for PI3K-axis co-alterations and *APC*+*PTEN*, may carry greater prognostic information than mutation burden alone.

*MET* was the clearest cohort-dependent signal. It was associated with lower hazard in the World cohort but trended adversely in North Sweden, alongside different variant spectra: X1010_splice and X963_splice predominated globally, whereas p.Thr992Ile was common in North Sweden. The regional excess of p.Thr992Ile raises the possibility of a population-enriched germline polymorphism rather than a recurrent somatic driver [19]. *MET* co-mutation with *EGFR* or TP53 may, nevertheless, modify prognosis through parallel pathway activation, genomic instability or treatment resistance [28–30].

Other enriched pairs, including CDKN2A+FBXW7, suggested worse survival but remained underpowered. Rare co-mutations will require larger series for definitive interpretation. Similarly, negatively enriched pairs such as *EGFR*+CDKN2A were uncommon, and any survival trends remain hypothesis-generating.

Limitations of this study include the constraints of a 67-gene panel, small co-mutant groups and incomplete stage data in the validation cohort. Progression-free survival (PFS) for specific treatments in the North Swedish cohort was not possible to evaluate due to lack of treatment data. Biopsies for the North Sweden cohort were obtained at diagnosis, and sequence data were obtained before first line treatment. Smoking and age shape the mutational landscape of NSCLC and may contribute to the co-mutation and survival patterns observed; our multivariable models adjust for these factors for the selected pairs, whereas the enrichment and survival screens were unadjusted, and the survival screen used uncorrected *p*-values and should be regarded as exploratory. These findings should be validated in larger cohorts with broader panels. Reporting *EGFR* and *KRAS* co-mutations appears clinically relevant today, whereas other co-mutational patterns require further validation.

In conclusion, our study demonstrates that co-mutation profiling reveals pathway-level interactions driving survival heterogeneity in ways that individual mutation burden cannot capture. *EGFR* emerges as a highly co-mutation-dependent driver gene. *KRAS*+STK11 and *APC*+*PTEN* were amongst the most reproducibly enriched and prognostically relevant pairs across both cohorts, reinforcing that co-selected combinations at pathway interfaces carry genuine survival consequences. Our data indicate that medium-sized panel profiling reveals more information than the presence of druggable driver genes alone [28]. The prognostic and predictive information already embedded in current panel data may help further refine personalised treatment.

## Supplementary Material



## Data Availability

The Swedish patient-level data underlying this study are held by the National Lung Cancer Registry and Clinical Genomics Umeå. Owing to ethical and privacy restrictions, these data are not publicly available but may be obtained from the corresponding author upon reasonable request, subject to ethical approval from the relevant Swedish Regional Ethics Review Board. The cBioPortal-derived data used for the validation cohort are freely available through the published studies cited [13–17].
