# Supplementary material for: Co-mutation patterns and survival associations in lung cancer: a comparative analysis of Swedish and global cohorts
Source: Acta Oncol. 2026 Oct 9;65:46330. doi: 10.2340/1651-226X.2026.46330 (PMC13660355; doi:10.2340/1651-226X.2026.46330)
Supplement: Supplementary file 1 [file AO-65-46330-s1.pdf]

Supplementary material has been published as submitted. It has not been copyedited, or typeset by Acta Oncologica

Supplementary Material

Co-Mutation Patterns and Survival Associations in Lung Cancer: A Comparative Analysis of Swedish and Global Cohorts

Karlowatz et al.

Supplementary Figure Legends

Supplementary Figures

Supp Fig
1A

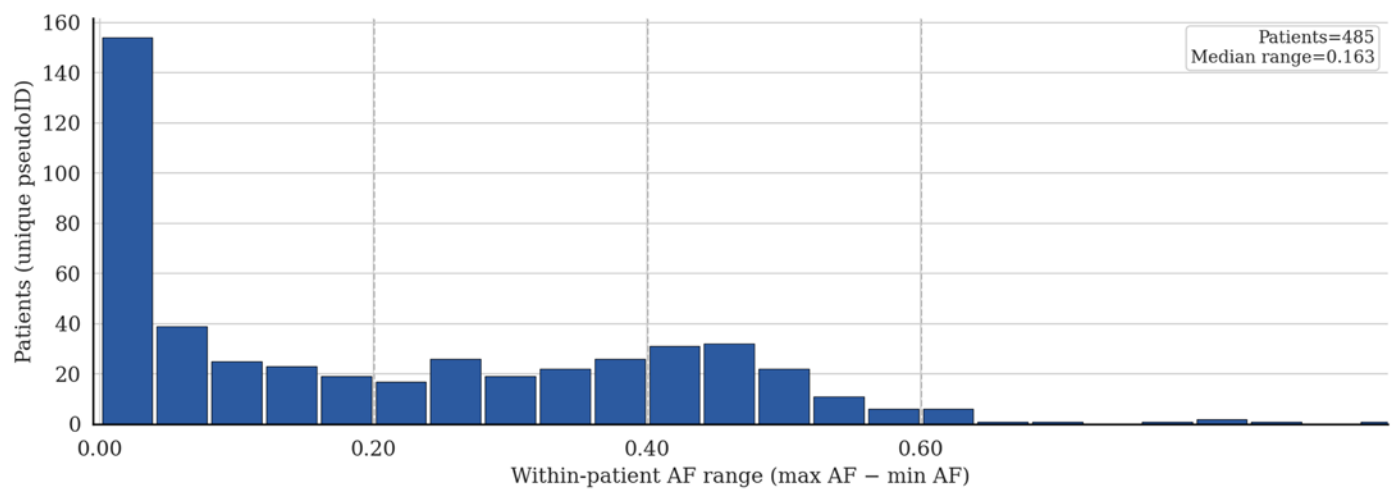

Supp Fig
1B

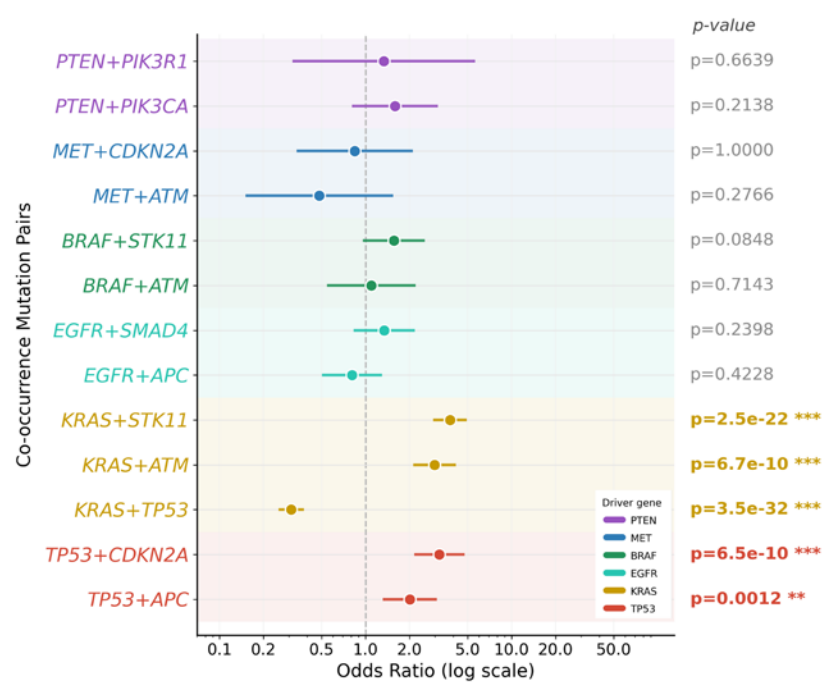

Supp Fig
1C

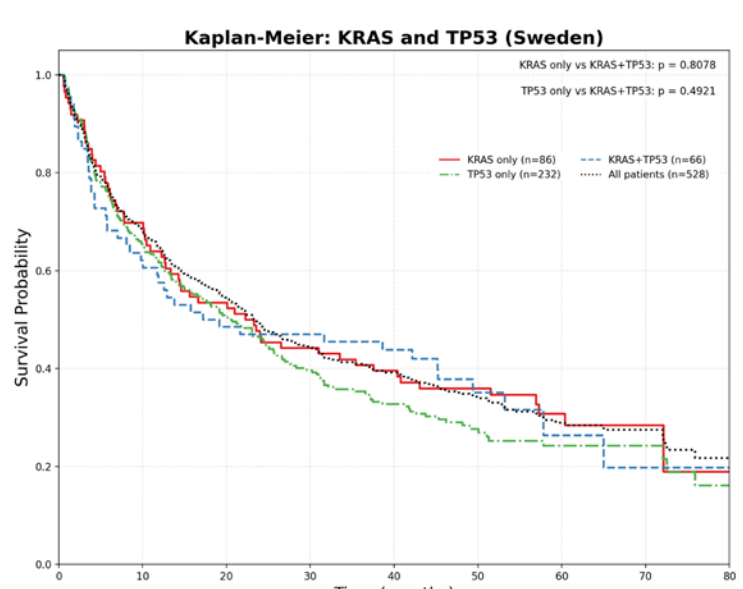

	0	12	24	36	48	60	72	80
KRAS only	86	55	40	35	29	13	3	1
TP53 only	232	143	107	82	46	20	9	4
KRAS+TP53	66	38	31	30	16	4	2	1
All patients	528	341	259	216	133	51	20	11

Supp Fig
1D

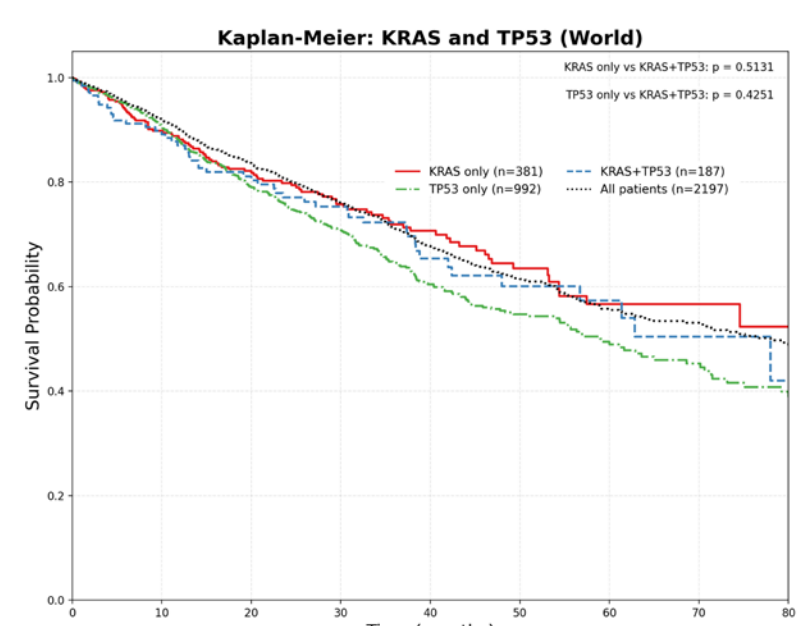

	0	12	24	36	48	60	72	80
KRAS only	381	260	195	120	72	29	14	11
TP53 only	992	591	436	284	172	96	56	44
KRAS+TP53	187	124	94	58	30	17	8	5
All patients	2197	1480	1131	737	452	244	144	112

Supplementary Figure 1A. Within-patient allele-frequency (AF) range in the North Sweden cohort. The within-patient AF range was defined as the maximum minus minimum AF across variants with AF ≥0.05 per patient and is shown as a histogram (bin width 0.04). The cohort included 484 patients with at least one variant meeting the AF threshold, and the median within-patient AF range was 0.163.

Supplementary Figure 1B. Co-mutation odds ratios in the World cohort (n = 2,239). Each point represents a gene pair with Fisher's exact P < 0.05; odds ratios (log scale) with 95% confidence intervals are shown, and the dashed line indicates an odds ratio of 1.

Supplementary Figure 1C. KRAS and TP53 co-mutation survival in the North Sweden cohort (n = 528). Kaplan–Meier estimates of overall survival are shown for KRAS-only, TP53-only, KRAS+TP53, and all patients; tick marks indicate censoring and numbers at risk are shown over time (months).

Supplementary Figure 1D. KRAS and TP53 co-mutation survival in the World cohort (n = 2,197 with overall-survival data). Kaplan–Meier curves and risk tables are shown as in Supplementary Figure 1C.

Supp
Fig 2A

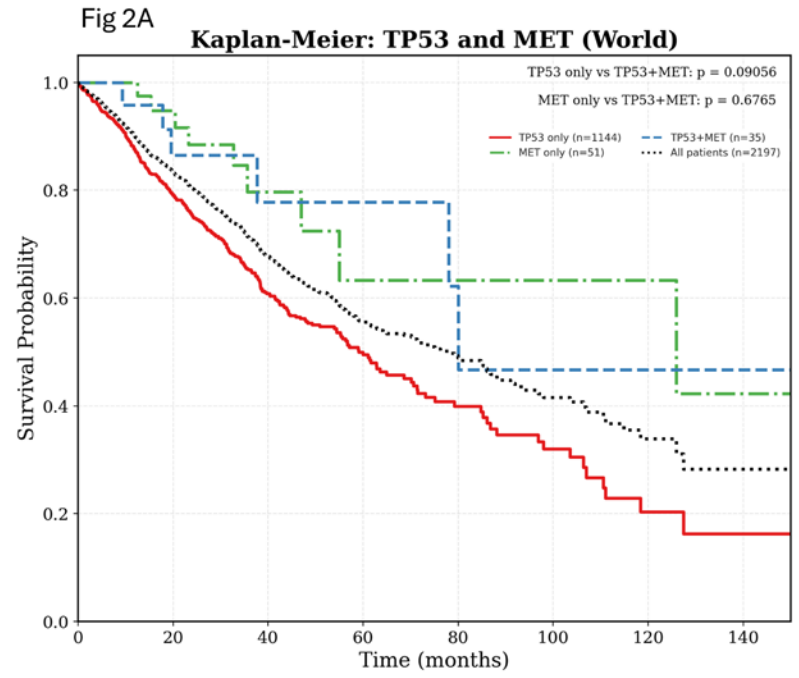

	0	12	24	36	48	60	72	84	96	108	120	132
TP53 only	1144	693	513	332	196	107	59	41	27	14	6	4
MET only	51	40	27	16	10	6	4	4	4	4	3	2
TP53+MET	35	22	17	10	6	6	5	3	2	1	1	1
All patients	2197	1480	1131	737	452	244	144	101	64	41	18	9

Supp
Fig 2B

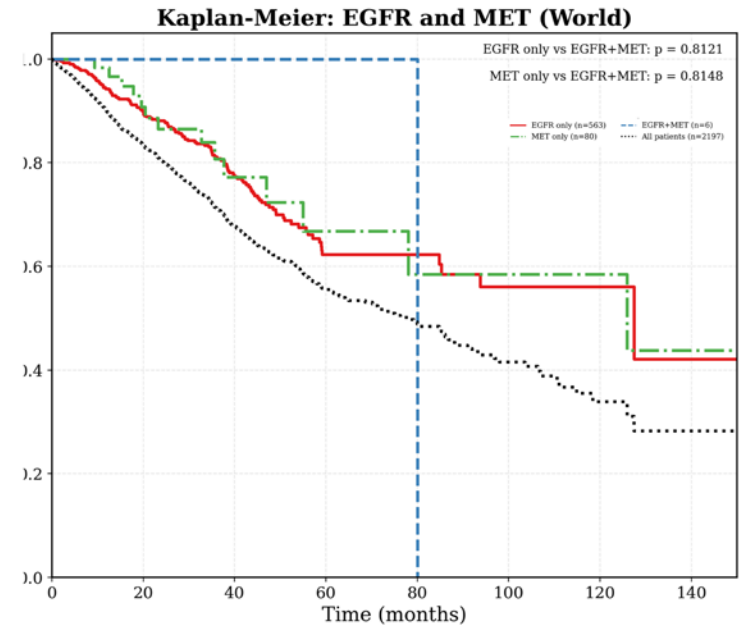

	0	12	24	36	48	60	72	84	96	108	120	132
EGFR only	563	438	370	252	151	75	50	34	21	13	7	2
MET only	80	57	39	24	15	11	8	7	6	5	4	3
EGFR+MET	6	5	5	2	1	1	1	0	0	0	0	0
All patients	2197	1480	1131	737	452	244	144	101	64	41	18	9

Supp
Fig 3A

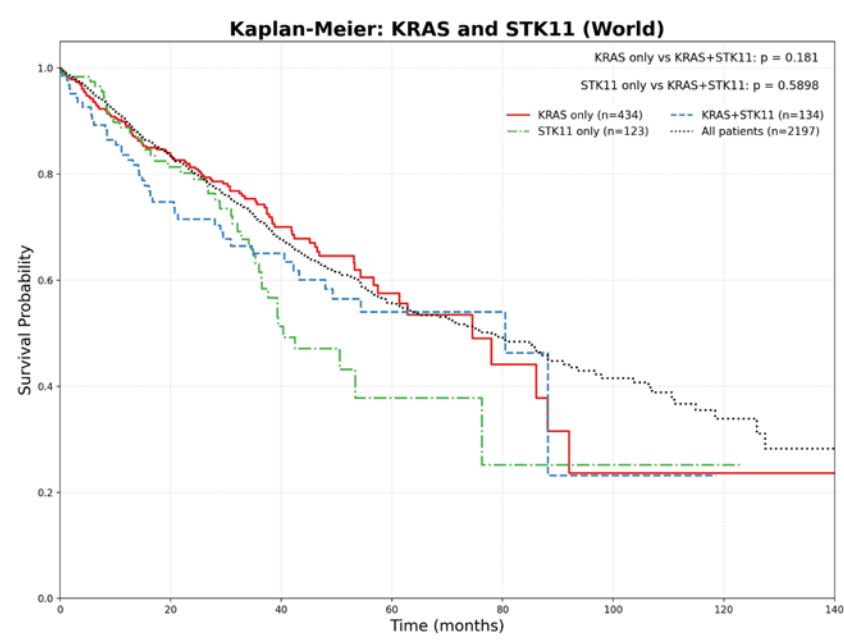

KRAS only	434	295	229	135	69	31	15	8	3	2	2	1
STK11 only	123	89	67	40	17	6	3	2	2	2	1	0
KRAS+STK11	134	89	60	43	33	15	7	5	1	1	0	0
All patients	2197	1480	1131	737	452	244	144	101	64	41	18	9

Supp
Fig3B

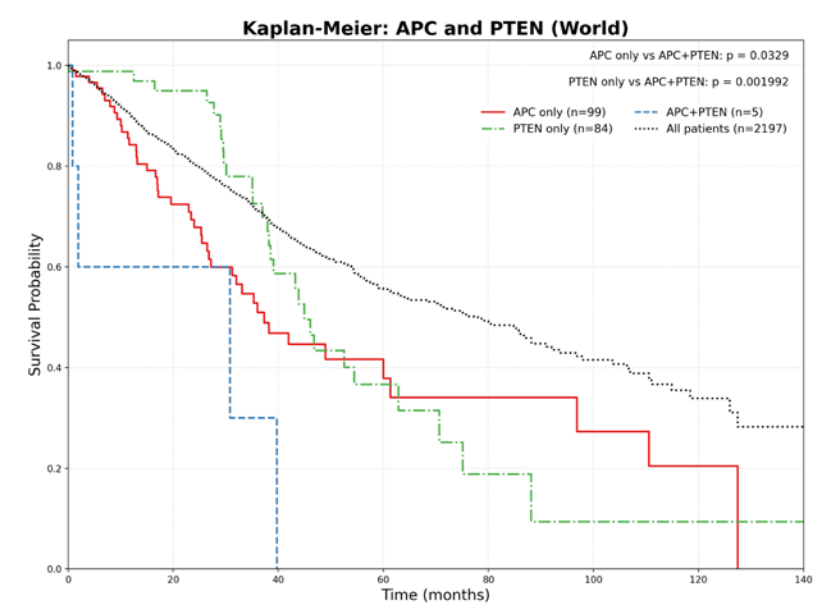

	0	12	24	36	48	60	72	84	96	108	120	132
APC only	99	66	45	28	16	11	6	6	5	4	2	0
PTEN only	84	53	44	27	14	9	4	2	1	1	1	1
APC+PTEN	5	2	2	1	0	0	0	0	0	0	0	0
All patients	2197	1480	1131	737	452	244	144	101	64	41	18	9

Supp Fig
3C

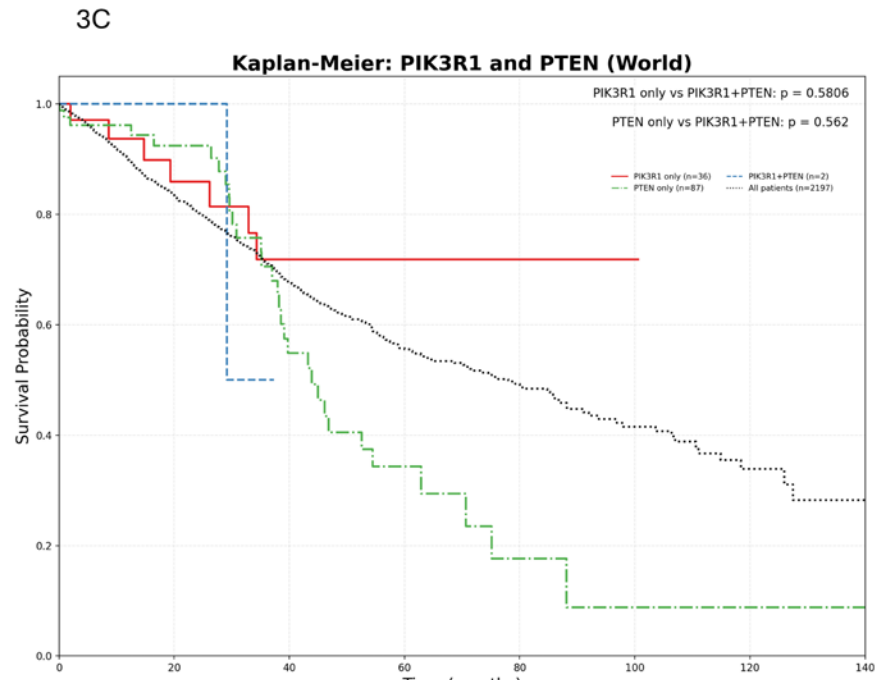

	0	12	24	36	48	60	72	84	96	108	120	132
PIK3R1 only	36	26	20	14	11	4	2	1	1	0	0	0
PTEN only	87	53	44	27	14	9	4	2	1	1	1	1
PIK3R1+PTEN	2	2	2	1	0	0	0	0	0	0	0	0
All patients	2197	1480	1131	737	452	244	144	101	64	41	18	9

Supp
Fig 3D

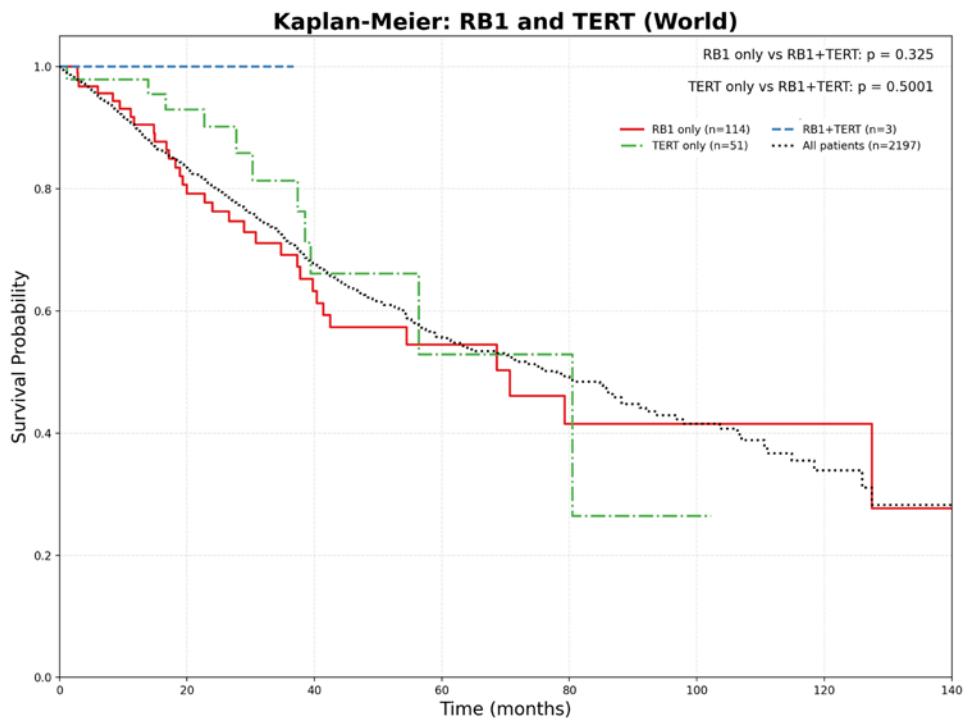

	0	12	24	36	48	60	72	84	96	108	120	132
RB1 only	19	14	11	7	6	2	1	1	0	0	0	0
TP53 only	1081	657	485	313	184	99	54	36	21	10	4	3
RB1+TP53	98	58	45	29	18	14	10	8	8	5	3	2
All patients	2197	1480	1131	737	452	244	144	101	64	41	18	9

Supp
Fig 3E

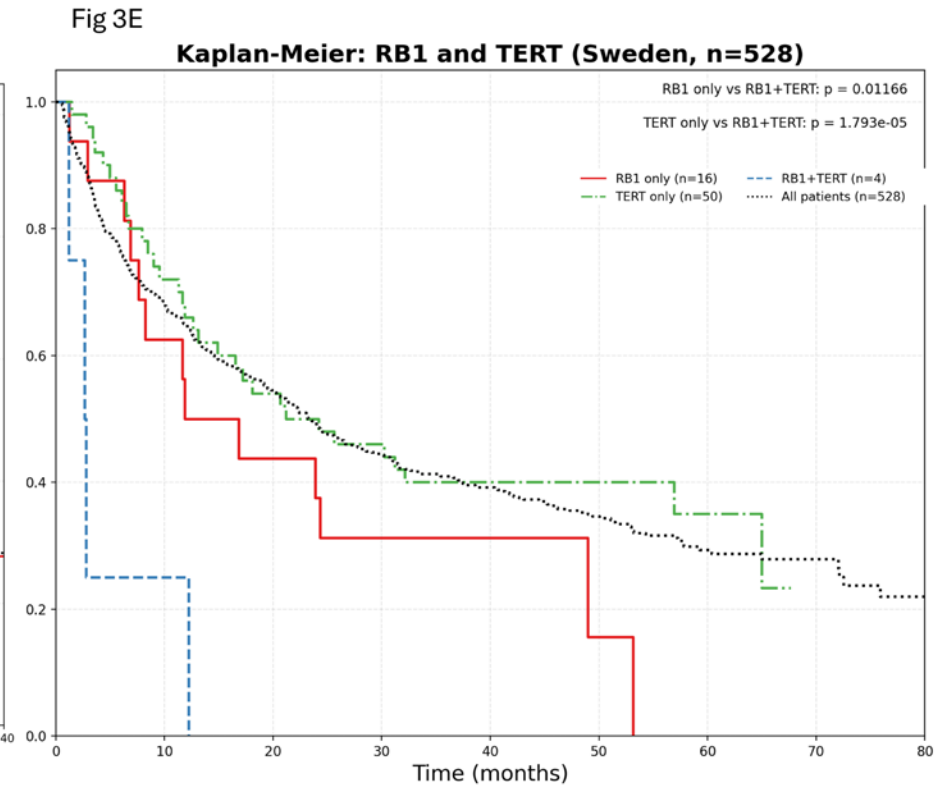

	0	12	24	36	48	60	72	80
RB1 only	16	8	6	5	2	0	0	0
TERT only	50	33	25	20	12	4	0	0
RB1+TERT	4	1	0	0	0	0	0	0
All patients	528	341	259	216	133	51	20	11

Supp
Fig 3F

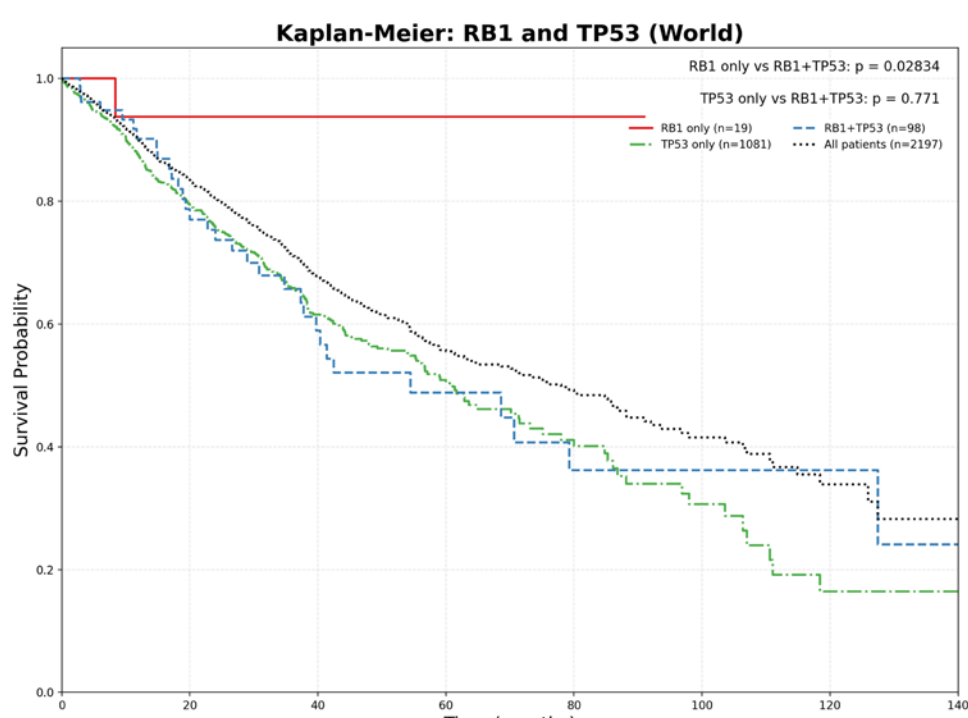

	0	12	24	36	48	60	72	84	96	108	120	132
RB1 only	19	14	11	7	6	2	1	1	0	0	0	0
TP53 only	1081	657	485	313	184	99	54	36	21	10	4	3
RB1+TP53	98	58	45	29	18	14	10	8	8	5	3	2
All patients	2197	1480	1131	737	452	244	144	101	64	41	18	9

Supp
Fig 3G

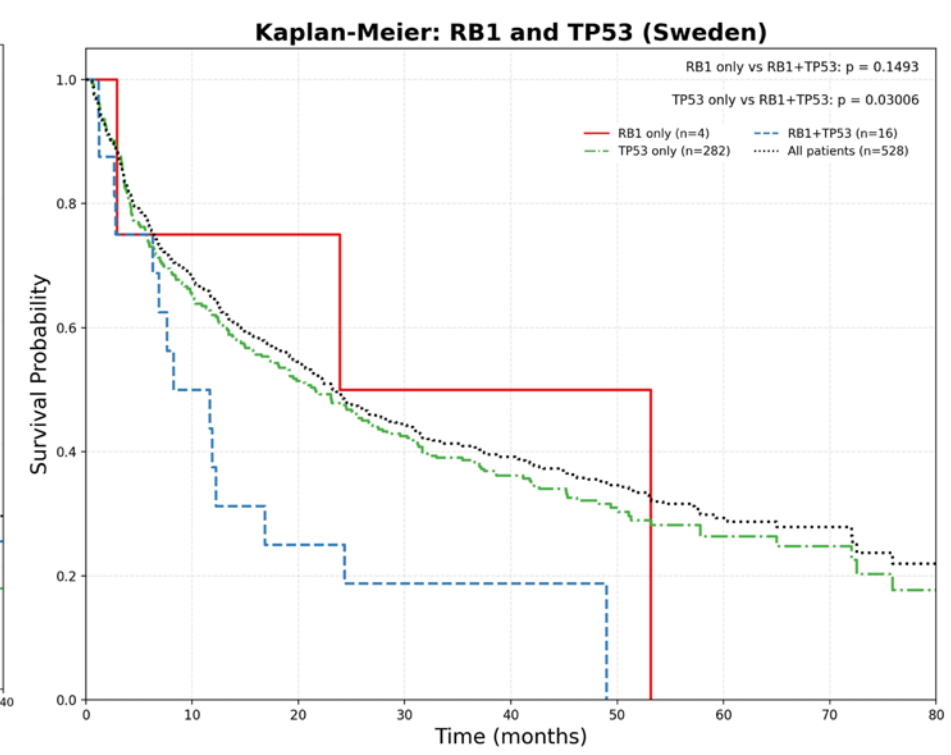

	0	12	24	36	48	60	72	80
RB1 only	4	3	2	2	1	0	0	0
TP53 only	282	175	134	109	61	24	11	5
RB1+TP53	16	6	4	3	1	0	0	0
All patients	528	341	259	216	133	51	20	11

Supp
Fig 3H

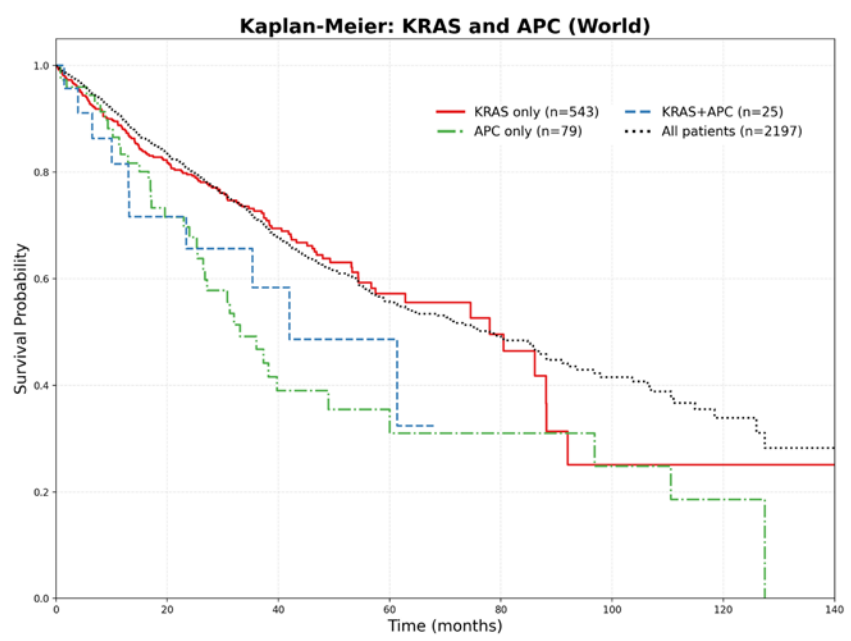

	0	12	24	36	48	60	72	84	96	108	120	132
KRAS only	543	367	278	170	98	43	22	13	4	3	2	1
APC only	79	51	36	21	12	8	6	6	5	4	2	0
KRAS+APC	25	17	11	8	4	3	0	0	0	0	0	0
All patients	2197	1480	1131	737	452	244	144	101	64	41	18	9

Supp
Fig 3I

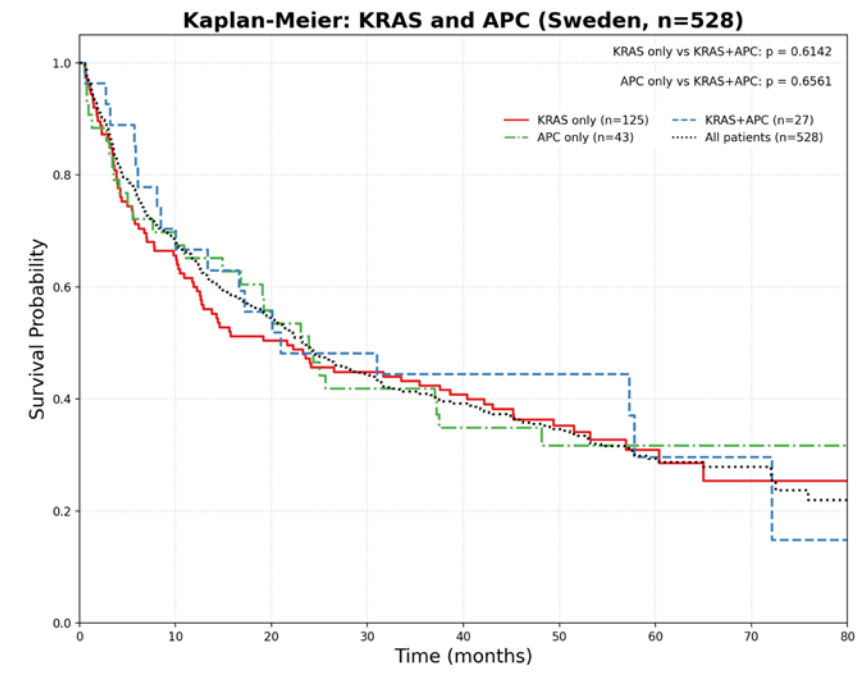

	0	12	24	36	48	60	72	80
KRAS only	125	75	58	53	36	13	3	1
APC only	43	28	21	18	11	5	2	2
KRAS+APC	27	18	13	12	9	4	2	1
All patients	528	341	259	216	133	51	20	11

Supp
Fig 3J

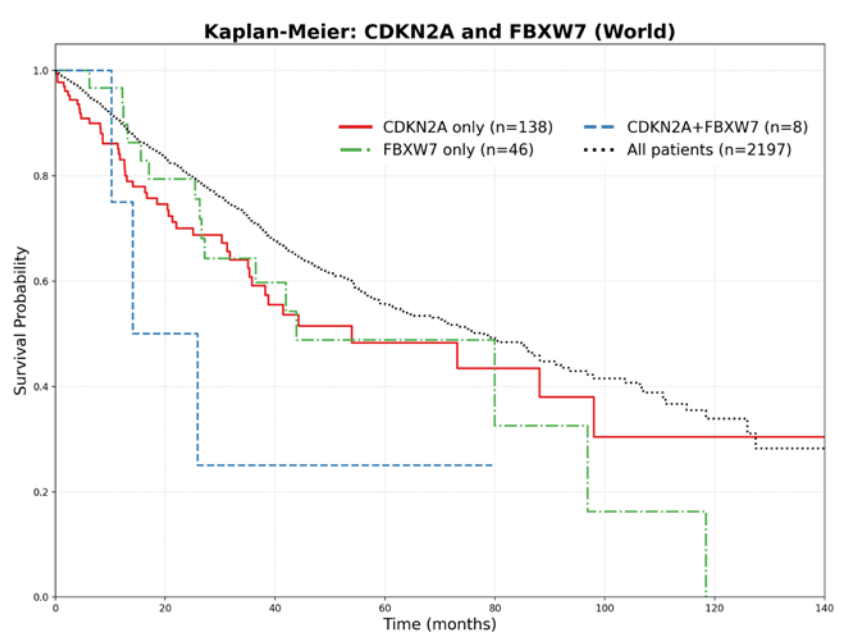

	0	12	24	36	48	60	72	84	96	108	120	132
CDKN2A only	138	81	58	36	20	13	10	8	7	4	1	1
FBXW7 only	46	28	22	14	8	5	3	2	2	1	0	0
CDKN2A+FBXW7	8	3	2	1	1	1	1	0	0	0	0	0
All patients	2197	1480	1131	737	452	244	144	101	64	41	18	9

Supp
Fig 3K

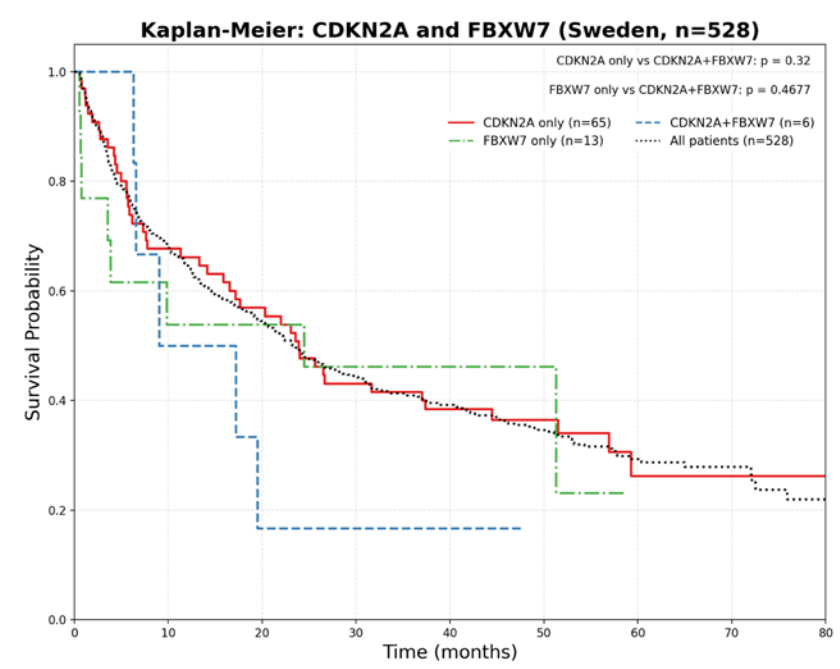

	0	12	24	36	48	60	72	80
CDKN2A only	65	43	31	27	17	6	3	1
FBXW7 only	13	7	7	6	2	0	0	0
CDKN2A+FBXW7	6	3	1	1	0	0	0	0
All patients	528	341	259	216	133	51	20	11

Supp
Fig 3L

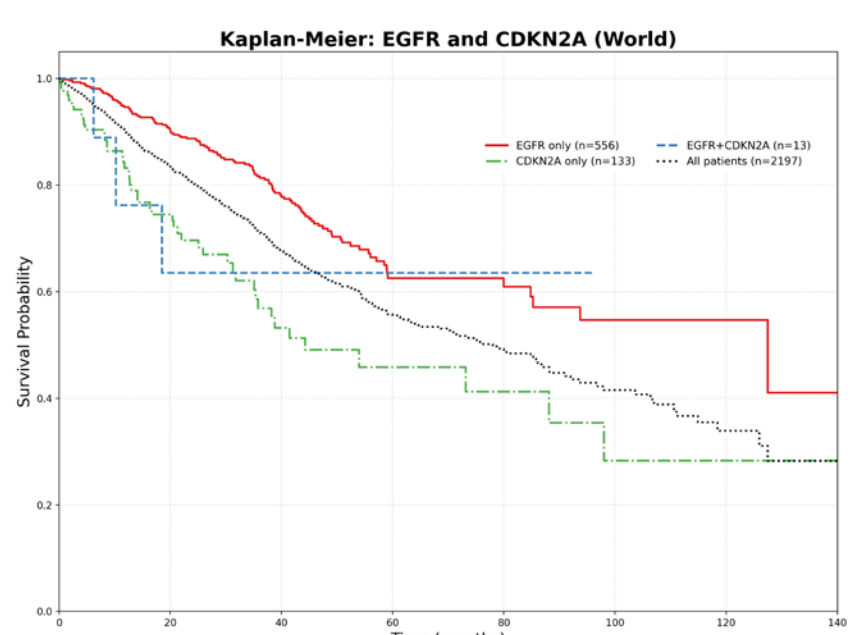

	0	12	24	36	48	60	72	84	96	108	120	132
EGFR only	556	437	370	250	150	75	50	33	20	13	7	2
CDKN2A only	133	78	55	33	19	13	10	7	6	4	1	1
EGFR+CDKN2A	13	6	5	4	2	1	1	1	1	0	0	0
All patients	2197	1480	1131	737	452	244	144	101	64	41	18	9

Supp
Fig 3M

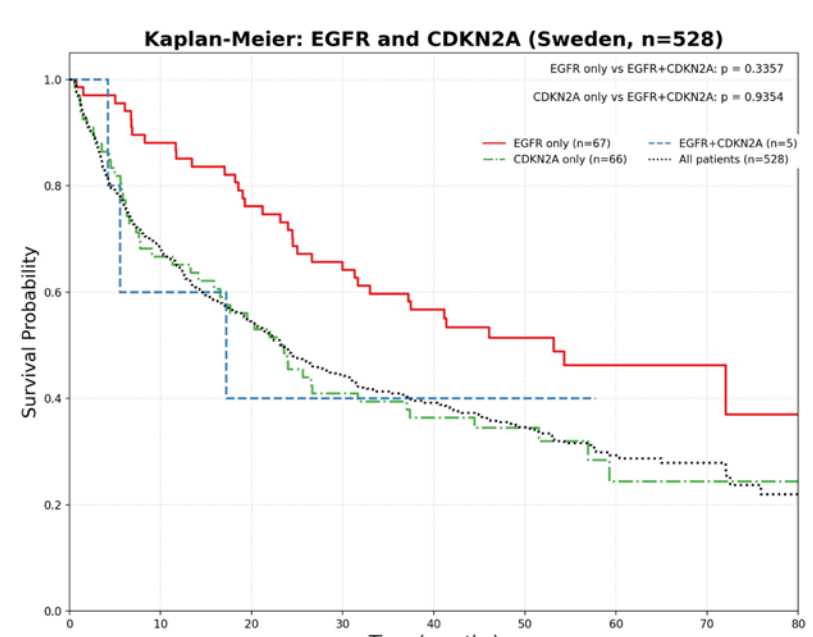

	0	12	24	36	48	60	72	80
EGFR only	67	57	49	40	24	13	5	3
CDKN2A only	66	43	30	26	16	6	3	1
EGFR+CDKN2A	5	3	2	2	1	0	0	0
All patients	528	341	259	216	133	51	20	11

Supp
Fig 3N

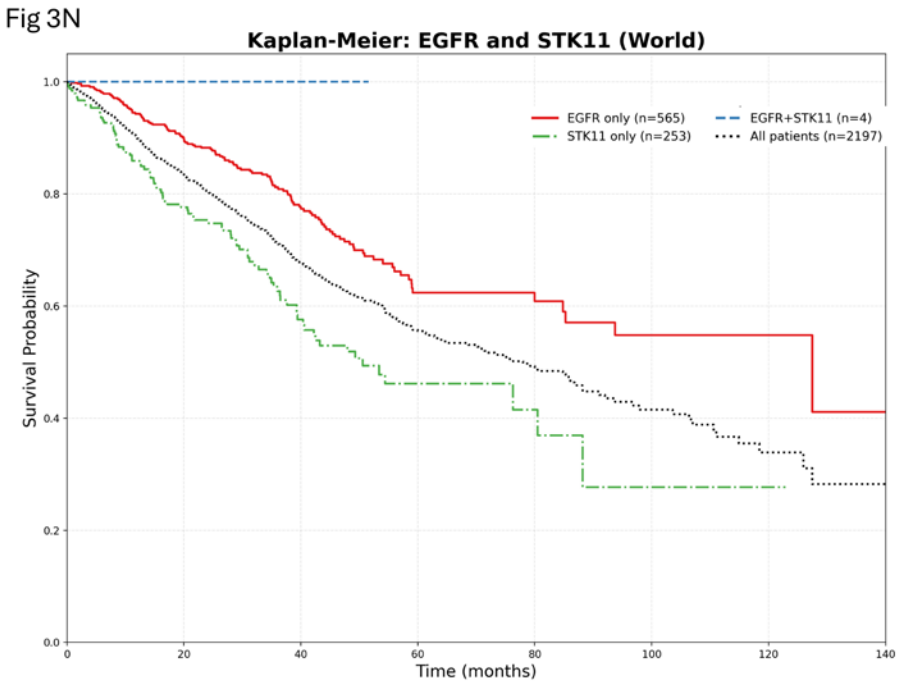

	0	12	24	36	48	60	72	84	96	108	120	132
EGFR only	565	440	372	251	151	76	51	34	21	13	7	2
STK11 only	253	175	124	80	49	21	10	7	3	3	1	0
EGFR+STK11	4	3	3	3	1	0	0	0	0	0	0	0
All patients	2197	1480	1131	737	452	244	144	101	64	41	18	9

Supp
Fig 3O

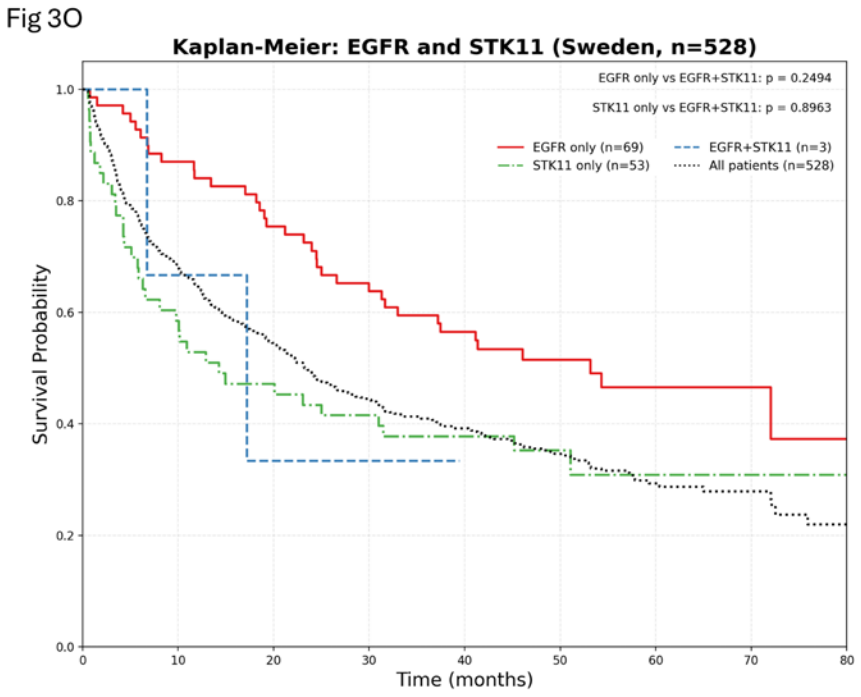

	0	12	24	36	48	60	72	80
EGFR only	69	58	50	41	25	13	5	3
STK11 only	53	28	23	20	11	3	1	1
EGFR+STK11	3	2	1	1	0	0	0	0
All patients	528	341	259	216	133	51	20	11

Supp
Fig 4A

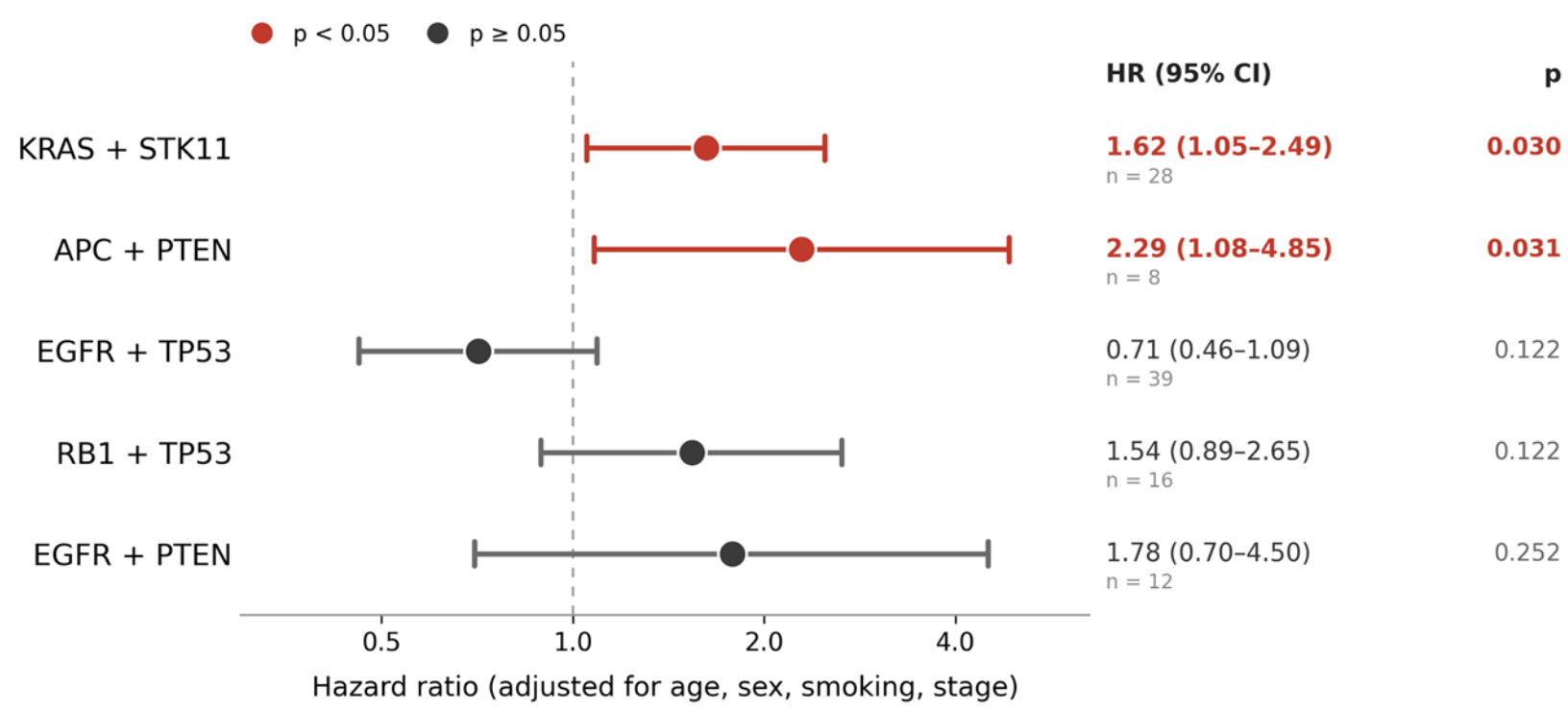

Supp
Fig 4B

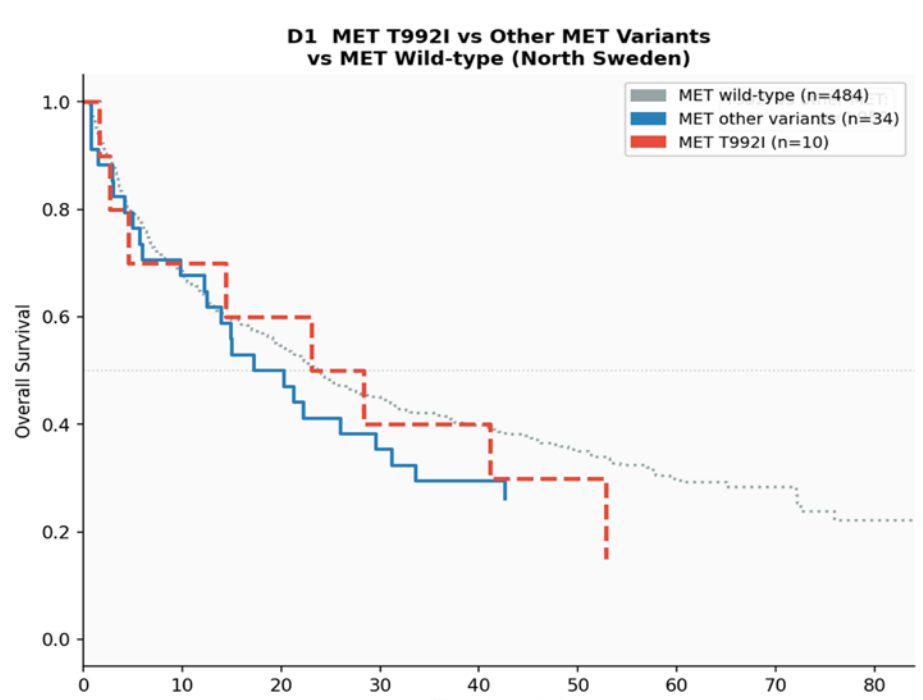

Supp
Fig 4C

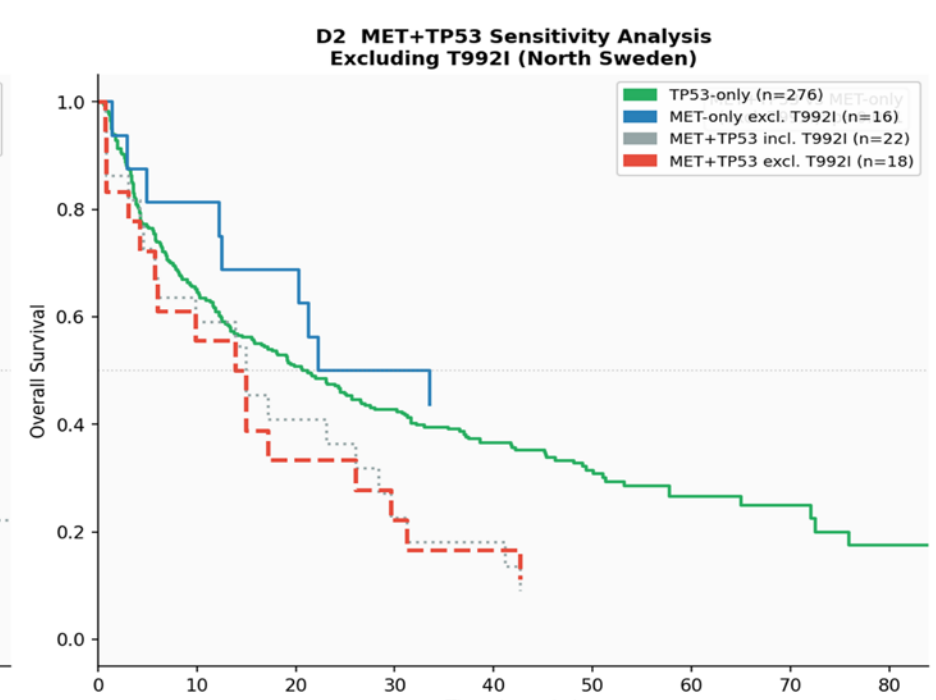

Supplementary Figure 2A. MET and TP53 co-mutation survival in the World cohort. Kaplan–Meier estimates of overall survival are shown for TP53-only, MET-only, TP53+MET, and all patients (time in months; tick marks indicate censoring).

Supplementary Figure 2B. MET and EGFR co-mutation survival in the World cohort. Kaplan–Meier curves and risk tables are shown for EGFR-only, MET-only, EGFR+MET, and all patients.

Supplementary Figures 3A–3O. Kaplan–Meier overall survival for additional co-mutation pairs in the World and North Sweden cohorts. Curves show gene-A-only, gene-B-only, co-mutated (A+B), and all patients; P values are from two-sided log-rank tests. Panels: 3A, KRAS and STK11 (World); 3B, APC and PTEN (World); 3C, PIK3R1 and PTEN (World); 3D, KRAS and APC (World); 3E, KRAS and APC (North Sweden); 3F, RB1 and TP53 (World); 3G, RB1 and TP53 (North Sweden); 3H, EGFR and KRAS (World); 3I, EGFR and KRAS (North Sweden); 3J, CDKN2A and FBXW7 (World); 3K, CDKN2A and FBXW7 (North Sweden); 3L, EGFR and CDKN2A (World); 3M, EGFR and CDKN2A (North Sweden); 3N, EGFR and STK11 (World); 3O, EGFR and STK11 (North Sweden).

Supplementary Figure 4A. Multivariable Cox proportional-hazards models in the North Sweden cohort (n = 528). Hazard ratios for selected co-mutation pairs (versus double wild-type) are shown with 95% confidence intervals, adjusted for age, sex, smoking history, and stage; P values are from Wald tests.

Supplementary Figure 4B. MET T992I sensitivity analysis in the North Sweden cohort. Kaplan–Meier overall survival is shown by MET variant class (T992I, other MET variants, and MET wild-type).

Supplementary Figure 4C. Sensitivity analysis for MET+TP53 in the North Sweden cohort excluding MET T992I. Kaplan–Meier curves compare MET+TP53 including versus excluding T992I to evaluate whether the association persists after removing a putative germline-enriched variant.

Supplementary Tables

Supplementary Table 1. Characteristics of the North Sweden and World validation cohorts. The World cohort was assembled from five publicly available datasets accessed via cBioPortal (June 2024). Median overall survival (OS) was estimated by the Kaplan–Meier method. Stage data were unavailable for the MSK-IMPACT and NCI never-smoker cohorts.

Study (reference)	N (with OS)	Median age (yr)	Median OS (mo)	Sequencing platform	Patient selection and context
**North Sweden NSCLC Registry (2010–2021)**	528 (528)	72	23.3	67-gene Archer VariantPlex targeted NGS	All lung cancer patients sequenced on regional molecular pathology platform, North Sweden; stages I–IV; 525/528 with stage data.
**TCGA Lung Adenocarcinoma (Cancer Genome Atlas, 2014)** *Nature 2014;511:543*	925 (900)	67	8.3	Whole-exome sequencing (Illumina HiSeq)	Resected primary lung adenocarcinoma, multi-institutional (North America); stages I–IV; 918/925 with stage data.
**MSK-IMPACT Lung Cancer (Zehir et al., 2017)** *Nat Med 2017;23:703*	896 (896)	—	30.6	MSK-IMPACT targeted panel (410–505 genes)	Consecutive lung cancer patients at Memorial Sloan Kettering; mixed real-world treatment; stage data unavailable in cBioPortal release.
**OncoSG Lung Adenocarcinoma (Chua et al., 2021)** *Clin Cancer Res 2021;27:6354*	290 (287)	64	36.9	Targeted hybrid-capture sequencing	EGFR-mutated NSCLC, Singapore; enriched for T790M-negative post-progression specimens; predominantly EGFR TKI-treated.
**NCI Never-Smoker Lung Cancer (Zhang et al., 2021)** *Nat Genet 2021;53:1348*	128 (114)	68	43.5	WGS / WES (multi-centre)	Never-smoker lung cancer, international cohort (Asia, Europe, North America); stage data unavailable.

N = total patients included in co-mutation analysis; values in parentheses are patients with overall survival data available. OS = overall survival, estimated by Kaplan–Meier method. Dash (—) indicates data not available in the cBioPortal release. All World cohort datasets accessed via cBioPortal (www.cbioportal.org, June 2024). MSK-IMPACT 2020 and 2023 releases were combined after deduplication. NGS = next-generation sequencing; WES = whole-exome sequencing; WGS = whole-genome sequencing; TKI = tyrosine kinase inhibitor.

Supplementary Table 2. Mutation frequencies for all 67 panel genes in the North Sweden and World cohorts. Percentages are of total patients per cohort (North Sweden n = 528; World n = 2,239). Sorted by North Sweden frequency.

Gene	North Sweden n (%)	World n (%)
**TP53**	298 (56.4%)	1200 (53.6%)
**KRAS**	152 (28.8%)	578 (25.8%)
**EGFR**	72 (13.6%)	579 (25.9%)
**CDKN2A**	71 (13.4%)	147 (6.6%)
**APC**	70 (13.3%)	104 (4.6%)
**ATM**	57 (10.8%)	148 (6.6%)
**STK11**	56 (10.6%)	261 (11.7%)
**TERT**	54 (10.2%)	55 (2.5%)
**MET**	44 (8.3%)	89 (4.0%)
**PIK3CA**	38 (7.2%)	167 (7.5%)
**SMAD4**	33 (6.2%)	79 (3.5%)
**PTEN**	27 (5.1%)	90 (4.0%)
**BRAF**	22 (4.2%)	126 (5.6%)

**RB1**	20 (3.8%)	118 (5.3%)
**FBXW7**	19 (3.6%)	54 (2.4%)
**CDH1**	17 (3.2%)	23 (1.0%)
**NOTCH1**	16 (3.0%)	94 (4.2%)
**PIK3R1**	16 (3.0%)	38 (1.7%)
**FGFR3**	16 (3.0%)	21 (0.9%)
**KDR**	15 (2.8%)	117 (5.2%)
**RET**	12 (2.3%)	66 (2.9%)
**ERBB4**	11 (2.1%)	140 (6.3%)
**FOXL2**	11 (2.1%)	16 (0.7%)
**ERBB2**	10 (1.9%)	83 (3.7%)
**SMO**	10 (1.9%)	37 (1.7%)
**ABL1**	10 (1.9%)	30 (1.3%)
**DDR2**	9 (1.7%)	57 (2.5%)
**CTNNB1**	8 (1.5%)	69 (3.1%)
**JAK3**	8 (1.5%)	58 (2.6%)
**MLH1**	8 (1.5%)	21 (0.9%)
**IDH1**	8 (1.5%)	12 (0.5%)
**MAP2K1**	7 (1.3%)	20 (0.9%)
**HNF1A**	6 (1.1%)	25 (1.1%)
**VHL**	6 (1.1%)	6 (0.3%)
**ALK**	5 (0.9%)	90 (4.0%)
**MYCN**	5 (0.9%)	14 (0.6%)
**HRAS**	5 (0.9%)	12 (0.5%)
**KIT**	4 (0.8%)	48 (2.1%)
**FGFR1**	4 (0.8%)	25 (1.1%)
**PTPN11**	4 (0.8%)	21 (0.9%)
**NRAS**	4 (0.8%)	19 (0.8%)
**SMARCB1**	4 (0.8%)	8 (0.4%)
**PDGFRA**	3 (0.6%)	97 (4.3%)
**ROS1**	3 (0.6%)	82 (3.7%)
**FLT3**	3 (0.6%)	54 (2.4%)
**CSF1R**	3 (0.6%)	44 (2.0%)
**EZH2**	3 (0.6%)	27 (1.2%)
**MPL**	3 (0.6%)	12 (0.5%)
**RHOA**	3 (0.6%)	11 (0.5%)
**MYC**	3 (0.6%)	10 (0.4%)
**CDK4**	3 (0.6%)	4 (0.2%)
**FGFR2**	2 (0.4%)	36 (1.6%)
**ERBB3**	2 (0.4%)	35 (1.6%)
**IDH2**	2 (0.4%)	5 (0.2%)
**ESR1**	1 (0.2%)	37 (1.7%)
**CCNE1**	1 (0.2%)	18 (0.8%)
**AKT1**	1 (0.2%)	17 (0.8%)
**SRC**	1 (0.2%)	11 (0.5%)
**GNA11**	1 (0.2%)	7 (0.3%)
**AURKA**	1 (0.2%)	5 (0.2%)

Supplementary Table 3. Unadjusted univariable Cox proportional-hazards regression for overall survival. Mutated versus wild-type status for EGFR, MET, and TP53 in each cohort.

Cohort	Gene	Mutated (n)	Patients with OS (n)	HR (95% CI)	p-value
North Sweden	EGFR	72	528	0.55 (0.40–0.77)	0.00056
North Sweden	MET	44	528	1.21 (0.85–1.74)	0.289
North Sweden	TP53	298	528	1.30 (1.05–1.60)	0.015
World	EGFR	569	2197	0.58 (0.47–0.70)	4.78e-08
World	MET	86	2197	0.59 (0.36–0.97)	0.036
World	TP53	1179	2197	1.60 (1.36–1.88)	1.96e-08

Supplementary Table 4. Distribution of MET protein-altering variants in the North Sweden and World cohorts. Some patients carry two MET variants; patient counts therefore exceed cohort totals. Sorted by total count.

Variant	North Sweden (n)	World (n)	Total (n)
**X1010_splice**	6	19	25
**X963_splice**	1	12	13
**p.Thr992Ile**	10	0	10
**p.Asp1010His**	0	6	6
**p.Asp1010Tyr**	2	3	5
**p.Asp1010Asn**	3	1	4
**X1006_splice**	0	2	2
**X1007_splice**	0	2	2
**X621_splice**	0	2	2
**p.Arg970Cys**	2	0	2
**p.Asp1010fs**	0	2	2
**p.Glu168Asp**	2	0	2
**p.Tyr1003Phe**	0	2	2
**p.Val136Ile**	2	0	2
**Not available**	0	1	1
**X1009_splice**	0	1	1
**X861_splice**	0	1	1
**X911_splice**	0	1	1
**p.Ala1005Ilefs*19**	0	1	1
**p.Ala179Ser**	0	1	1
**p.Ala179Thr**	1	0	1
**p.Ala364Thr**	1	0	1
**p.Arg1336Trp**	1	0	1
**p.Arg400Ser**	1	0	1
**p.Arg412His**	0	1	1
**p.Asn202Asp**	0	1	1
**p.Asn315Ser**	0	1	1
**p.Asn381Thrfs*3**	0	1	1
**p.Asp372Asn**	1	0	1
**p.Cys98Phe**	0	1	1
**p.Gln272Ter**	0	1	1
**p.Gln425Ter**	0	1	1
**p.Glu1314Gly**	0	1	1
**p.Glu293Lys**	0	1	1
**p.Gly1019Cys**	0	1	1
**p.Gly1090Ser**	1	0	1
**p.Gly577Val**	0	1	1
**p.Gly578Glu**	0	1	1
**p.Gly87Arg**	1	0	1
**p.His1094Arg**	0	1	1
**p.His1094Gln**	1	0	1
**p.His476Tyr**	0	1	1
**p.His60Arg**	1	0	1

Variant	North Sweden (n)	World (n)	Total (n)
**p.His60Gln**	1	0	1
**p.Ile11Val**	1	0	1
**p.Ile316Met**	1	0	1
**p.Ile565Met**	0	1	1
**p.Ile915Val**	0	1	1
**p.Leu1097Ser**	1	0	1
**p.Leu317Ile**	0	1	1
**p.Leu43Phe**	1	0	1
**p.Lys1259Asn**	1	0	1
**p.Lys902Ile**	0	1	1
**p.Lys957Thr**	0	1	1
**p.Pro1008Ala**	0	1	1
**p.Pro1008Argfs*5**	1	0	1
**p.Pro93Leu**	0	1	1
**p.Ser156Trp**	0	1	1
**p.Ser441Cys**	0	1	1
**p.Ser990Thr**	1	0	1
**p.Thr1096Ser**	0	1	1
**p.Thr230Met**	0	1	1
**p.Thr47Ser**	0	1	1
**p.Thr660Arg**	0	1	1
**p.Thr698Ser**	0	1	1
**p.Trp1249Arg**	0	1	1
**p.Trp1320Cys**	0	1	1
**p.Trp586Cys**	0	1	1
**p.Trp954Ter**	0	1	1
**p.Tyr1003Cys**	0	1	1
**p.Tyr1003Ter**	0	1	1
**p.Tyr1235Cys**	0	1	1
**p.Tyr1235His**	0	1	1
**p.Tyr321His**	0	1	1
**p.Tyr971Ter**	0	1	1
**p.Val81Leu**	1	0	1
**p.Val989Ile**	1	0	1

Supplementary Table 5. Co-mutation enrichment in the North Sweden and World cohorts using Fisher's exact test. OR > 1 indicates co-occurrence and OR < 1 mutual exclusivity; q-values are Benjamini-Hochberg adjusted. North Sweden pairs met minimum group-size criteria, and World columns show corresponding n, OR, direction, and FDR significance. *** q < 0.001; ** q < 0.01; * q < 0.05; n.s., not significant; —, not shown/available.

Co-mutation pair	NS n	NS OR	NS direction	NS p	NS q	NS FDR	World n	World OR	World direction	World FDR
KRAS+EGFR	5	0.16	Mutual exclusivity	2.2 x 10^-6	3.7 x 10^-5	***	8	0.03	Mutual exclusivity	***
KRAS+TP53	66	0.48	Mutual exclusivity	1.5 x 10^-4	0.002	**	188	0.31	Mutual exclusivity	***
EGFR+STK11	-	-	-	-	-	-	4	0.04	Mutual exclusivity	***
KRAS+STK11	28	2.81	Co-occurrence	4.7 x 10^-4	0.003	**	136	3.78	Co-occurrence	***
TP53+CDKN2A	51	2.17	Co-occurrence	0.005	0.015	*	114	3.2	Co-occurrence	***
EGFR+CDKN2A	5	0.44	Mutual exclusivity	0.094	0.123	n.s.	13	0.26	Mutual exclusivity	***
BRAF+KRAS	5	0.72	Mutual exclusivity	0.640	0.680	n.s.	12	0.29	Mutual exclusivity	***
EGFR+TP53	39	0.90	Mutual exclusivity	0.700	0.700	n.s.	263	0.64	Mutual exclusivity	***
EGFR+BRAF	-	-	-	-	-	-	13	0.31	Mutual exclusivity	***
PTEN+TP53	21	2.83	Co-occurrence	0.027	0.059	n.s.	68	2.78	Co-occurrence	***
PTEN+PIK3R1	5	10.12	Co-occurrence	7.1 x 10^-4	0.003	**	2	1.33	Co-occurrence	n.s.
CDKN2A+FBXW7	6	3.15	Co-occurrence	0.031	0.059	n.s.	8	2.56	Co-occurrence	*
APC+PTEN	8	2.98	Co-occurrence	0.018	0.051	n.s.	5	1.22	Co-occurrence	n.s.
KRAS+SMAD4	15	2.18	Co-occurrence	0.045	0.070	n.s.	17	0.78	Mutual exclusivity	n.s.
KRAS+ATM	24	1.95	Co-occurrence	0.029	0.076	n.s.	72	2.97	Co-occurrence	***
PTEN+PIK3CA	5	3.22	Co-occurrence	0.037	0.080	n.s.	10	1.59	Co-occurrence	n.s.
